# A Better Zn-Ion Storage Device: Recent Progress for Zn-Ion Hybrid Supercapacitors

**DOI:** 10.1007/s40820-022-00793-w

**Published:** 2022-02-23

**Authors:** Jialun Jin, Xiangshun Geng, Qiang Chen, Tian-Ling Ren

**Affiliations:** 1 School of Integrated Circuits and Beijing National Research Center for Information Science and Technology (BNRist), Tsinghua University, Beijing, 100084 People’s Republic of China; 2 College of Material Science and Engineering, Zhejiang University of Technology, Hangzhou, 310014 People’s Republic of China

**Keywords:** Zn-ion storage systems, Zn-ion hybrid supercapacitors, Carbon electrodes, Zinc anodes, Electrolytes

## Abstract

As a new generation of Zn-ion storage systems, Zn-ion hybrid supercapacitors (ZHSCs) garner tremendous interests recently from researchers due to the perfect integration of batteries and supercapacitors. ZHSCs have excellent integration of high energy density and power density, which seamlessly bridges the gap between batteries and supercapacitors, becoming one of the most viable future options for large-scale equipment and portable electronic devices. However, the currently reported two configurations of ZHSCs and corresponding energy storage mechanisms still lack systematic analyses. Herein, this review will be prudently organized from the perspectives of design strategies, electrode configurations, energy storage mechanisms, recent advances in electrode materials, electrolyte behaviors and further applications (micro or flexible devices) of ZHSCs. The synthesis processes and electrochemical properties of well-designed Zn anodes, capacitor-type electrodes and novel Zn-ion battery-type cathodes are comprehensively discussed. Finally, a brief summary and outlook for the further development of ZHSCs are presented as well. This review will provide timely access for researchers to the recent works regarding ZHSCs.

## Highlights

- The advances of electrode materials, energy storage mechanisms, electrolytes and applications for Zn-ion hybrid supercapacitors (ZHSCs) are comprehensively summarized.
- Recent progresses in ZHSCs are discussed by categorizing into two configurations of Zn//Cap and Cap//ZBC.
- Future opportunities and challenges for the development of ZHSCs are also elaborated.

## Graphical abstract

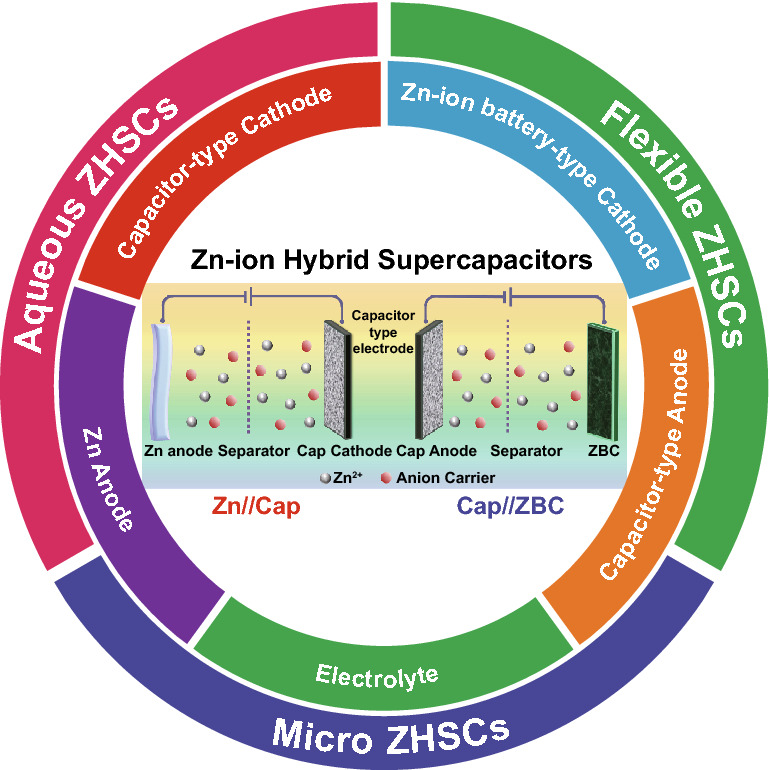

## Introduction

As numerous portable electronic devices and electric vehicles are popularized and widely used, energy storage systems (ESSs) with excellent electrochemical performance (e.g., long cycling lifetime and high capacity) are playing a highly vital role in modern society [1, 2]. Thus, various ESSs have been widely investigated and applied for commercial applications in recent years, such as metal-air batteries [3–5], aqueous batteries [6–10], supercapacitors [11–13] and others [14–22]. However, bottlenecks still exist in both batteries and supercapacitors, where the batteries have ultra-high energy density but suffer from the short cycling lifetime due to excessive redox reactions while the supercapacitors possess predominant power density and long cycling lifetime but support fewer capacity than batteries [23–28]. Recently, to integrate respective advantages of batteries and supercapacitors into one individual device, the concept of hybrid supercapacitors (HSCs) become quite popular where the battery-type electrodes with abundant redox reaction act as an energy source and the capacitor-type (Cap) electrodes with fast ionic conductivity act as a power source [29–33]. Significantly, it is believed that the well-designed configuration of hybrid supercapacitors can bridge the gap between batteries and supercapacitors, and make the best use of both devices characteristics, showing an optimum ESSs for social demands [34–40].

Generally, metal-ion HSCs are diverse based on the different charge carriers like lithium-ion (Li-ion) [37, 41–43], sodium-ion (Na-ion) [44–49], potassium-ion (K-ion) [50–52] and zinc-ion (Zn-ion) [34, 53]. These HSCs have been widely studied and enormous developments have been made in recent years, such as Li-ion hybrid supercapacitors [54–57] and Na-ion hybrid supercapacitors [58–61]. Nevertheless, compared with the divalent Zn-ion, alkaline metal-ions present some deficiencies in practical applications. For instance, the organic electrolytes employed in Li-ion batteries (LIBs) and the excessively active redox reactions between alkaline metals and water/air, which lead to serious safety risks, potential environmental pollutions and complex manufacturing processes [35, 62–65]. Against this background, Zn-ions show the smallest radius and highest electrode potential (− 0.76 V *vs*. standard hydrogen electrode, SHE) among the metal-ions mentioned above, enabling Zn-ions to be highly efficient and safe charge carriers for aqueous HSCs [66, 67]. Notably, the Zn-ion ESSs have already been developed for almost 200 years, many Zn-ion ESSs showed the decisive impact on ESSs researches and have been intensively utilized in electronic devices, such as Zn-air [68–70], Zn–Ni [71–73], Zn–Ag [74–76] and various aqueous Zn-ion batteries (ZIBs) [67, 77]. Nowadays, wide applications of portable mobile electronic devices give rise to stricter requirements on the energy density and cycling ability of ESSs [78–81]. Hence, recently reported Zn-ion hybrid supercapacitors (ZHSCs) have exhibited attractive prospects include superb energy density, better power density, long cycling lifetime and splendid applications in flexible and micro-scale devices [32, 53, 82, 83]. Although some researches on ZHSCs have been developed, this novel ESS is still in its infancy, a systematic-and-comprehensive review including the design theories of two electrode configurations and energy storage mechanisms is necessary, which can be instructional for future investigations of ZHSCs [35, 84].

**Scheme 1:**
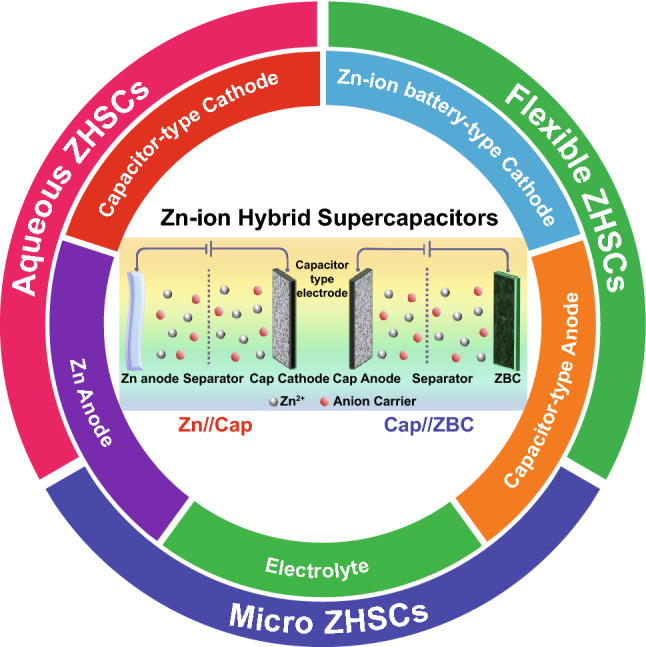
Summary of the aspects discussed in this review and schematic illustration of two configurations (Zn//Cap and Cap//ZBC) of ZHSCs

Herein, we systematically reviewed the recent progress in researches on ZHSCs. First, we classified the ZHSCs into two configurations according to the electrode materials and elaborated on the energy storage mechanisms of each configuration. As depicted in the Scheme 1, when the battery-type cathode or anode of ZIBs are substituted with Cap electrodes, the corresponding two configurations of ZHSCs are created. The first configuration of ZHSCs is constructed by the Zn anode and Cap cathode, while the second configuration of ZHSCs is assembled by Cap anode and Zn-ion battery-type cathodes (ZBC). For brevity, these two configurations of ZHSCs are named as Zn//Cap ZHSCs and Cap//ZBC ZHSCs, respectively. In addition, the synthesis strategies, typical characteristics and electrochemical performance of electrode materials in each configuration of ZHSCs are introduced, including Zn anodes, various Cap electrodes and novel ZBC. Significantly, the formation mechanisms of by-products are carefully concluded and three typical energy storage mechanisms of Cap electrodes are summarized. The effects of various Zn-based electrolytes and additive electrolytes on the Zn deposition and electrochemical performance of ZHSCs are emphasized. After that, we outlined the interesting application of ZHSCs for Zn-ion hybrid micro-supercapacitors (ZmSCs) and flexible ZHSCs devices. Finally, brief outlooks based on the recent ZHSCs progress are provided. We hope this review can attract more attention to this new generation Zn-ion ESS and promote its further development and even practical applications in modern society.

## Aqueous Zn-Ion Hybrid Supercapacitors

Due to the special electrode potential of Zn electrodes and suitable Zn-ion salt electrolytes, the aqueous system of ZHSCs can be easily achieved and further promote the development of eco-friendly ESSs. In this section, we will summarize the recently reported aqueous ZHSCs system of two electrode configurations, the corresponding design theory, electrochemical performance and the energy storage mechanisms of electrode materials. Subsequently, some workable synthesis strategies of each electrode are introduced. Note that the Cap electrodes will be discussed between the two electrode configurations of ZHSCs.

### Zn//Cap ZHSCs

**Fig. 1:**
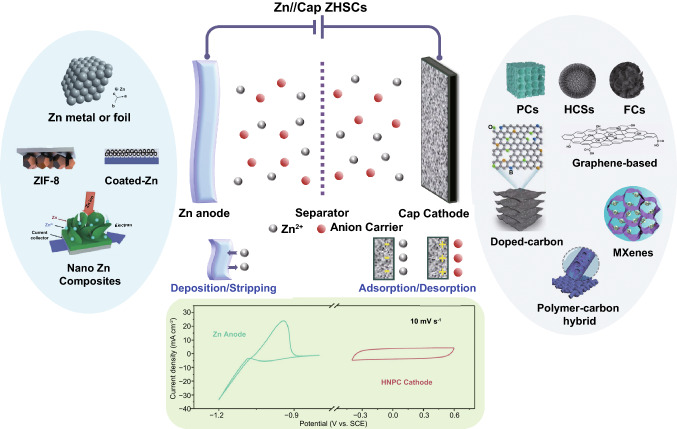
Schematic illustration (Zn anodes, Cap cathodes, energy storage mechanisms and CV behaviors) of Zn//Cap ZHSCs

The first electrode configuration of Zn//Cap ZHSCs is shown in Fig. 1, where Cap electrodes act as the cathode and power source, Zn foil or electrodeposited Zn composite electrodes act as the anode and energy source, and Zn salt solutions are employed as the electrolytes. To evaluate the energy storage mechanisms of Zn//Cap ZHSCs, the cyclic voltammetry (CV) curves of representative Cap cathode and Zn anode are depicted in Fig. 1 as well. The sharp redox peaks at − 1.2 V/− 0.94 V (*vs*. saturated calomel electrode, SCE) are detected on the Zn anode, corresponding to the depositing/stripping processes of Zn/Zn^2+^ redox reactions while the ideal rectangular shape is presented on the N-doped hierarchically porous carbon (HNPC) cathode (represent most Cap electrodes) [85]. Typically, the rectangular CV curve indicated that the physical adsorption/desorption reaction is the dominant process on the surface of Cap electrodes, resulting in an electric double-layer capacitance (EDLC). Although the designed prototype of ZHSC is quite novel, many obstacles appeared when constructing high-performance ZHSCs due to the limitations of various electrode materials. Hence, we reviewed some reported Zn anodes in the system of Zn//Cap ZHSCs and summarized feasible synthesis strategies for Zn anodes.

Zn anode, as the energy source of ZHSCs, proceeds with the depositing/stripping reaction of Zn/Zn^2+^ during charging/discharging process. Generally, the depositing/stripping reaction equation can be expressed by Eq. (2-1) [34, 85]:

2-1 $${\text{Zn }} \leftrightarrow {\text{ Zn}}^{{{2} + }} + {\text{ 2e}}^{ - }$$

Zn foil is a usual and suitable Zn anode material in the configuration of Zn//Cap ZHSCs owing to the advantages of abundant redox reactions, low flammability, flexibility and low cost [86, 87]. Even so, similar to lithium dendrites in LIBs, Zn dendrite also presented on the surface of bare Zn foil, which can be a potential safety hazard for electronic devices [88, 89]. Generally, the formation mechanism of Zn dendrite can be ascribed to the uneven distribution of Zn-ion contributed by the low-speed charging/discharging speed on the planar of two-dimensional (2D) Zn foil, which results in uneven nucleation and poor reversibility. The weeny tips on the pristine Zn foil can become the charge center of the electric field and further grow to Zn dendrites with the accumulation of charge. Although the formation of Zn dendrite can be suppressed by the fast charging/discharging property of well-designed ZHSCs, efforts should be devoted to thoroughly eliminating the Zn dendrite for the ESSs safety and promoting longevity of ZHSCs. Additionally, the hydrogen evolution induced by the H_2_O decomposition can greatly affect the depositing/stripping efficiency of flat Zn foil and increase the concentration of OH^−^ in electrolyte, which may lead to the formation of by-products on the surface of both Zn anode and Cap cathode [90]. Therefore, a well-designed Zn anode with fast and high-efficient properties is necessary to realize high-performance ZHSCs. In the following section, we will summarize some strategies from the reported Zn anodes in ZHSCs: (1) Zn foil coating protection; (2) Electrodeposited nanostructure Zn anode; (3) Recoverable Zn anode.

#### Zn Foil Coating Protection

According to the above analysis of Zn dendrite, bare Zn foil with the planar and smooth surface is unfit for long cycling charging/discharging process. The Zn foil//ZnSO_4_ (gel) //layered B, N co-doped porous carbon (BN-LDC) ZHSCs reported by Lu et al*.* can reach a high capacity of 127.7 mAh g^−1^ but only maintain capacity retention of 81.3% after 6500 cycles at 5 A g^−1^ [83]. Additionally, Sun et al*.* reported Zn foil//Ti_3_C_2_ ZHSCs with a high capacity of ~ 1 F cm^−2^ in the aqueous electrolyte of ZnSO_4_, however, the Zn foil//Ti_3_C_2_ ZHSCs presented an inferior cycling performance, where the retention decay to 78% of initial capacity after 4000 cycles at a higher current density of 10 A g^−1^ [91].

**Fig. 2:**
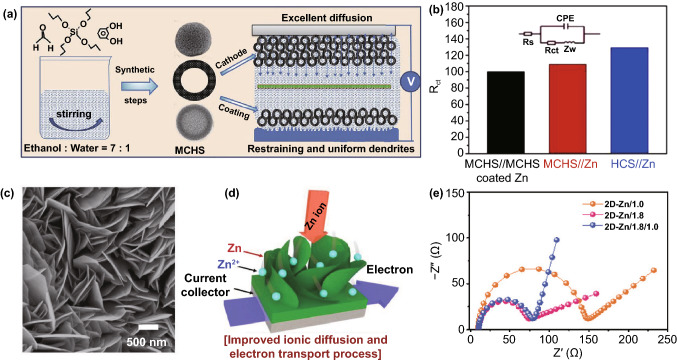
**a** Schematic illustration of the synthesis process of MCHSs and corresponding application in electrodes of ZHSCs. **b** Comparison of *R*_ct_ value for the three devices. Reproduced with permission from Ref. [92]. Copyright 2019, Elsevier. c SEM image and **d** schematic illustration of the hierarchically 2D-Zn anode. **e** Nyquist plots of different 2D-Zn anodes. Reproduced with permission from Ref. [53]. Copyright 2020, Wiley–VCH

By far, many dendrite-free Zn anodes of ZHSCs have been reported and one of the most promising strategies is the Zn foil coating protection, which can greatly stabilize and improve the performance of Zn anode by covering stable components with a rough surface. As shown in Fig. 2a, Liu et al*.* applied mesoporous carbon hollow spheres (MCHSs) as the external protective layer of Zn foil anode, and simultaneously, the MCHSs are also employed as the cathode materials of ZHSCs [92]. The MCHS-coated Zn foil remained smooth and porous after long cycling test while some spikes emerged on the surface of the bare Zn foil. Typically, the external MCHSs protective layer can restrain and uniform the Zn dendrite during Zn depositing/stripping process. As a result, the MCHS-coated Zn foil//MCHSs can deliver excellent cycling performance of 96% capacity retention after 10,000 cycles at a low current density of 1 A g^−1^, indicating the good stability of MCHS-coated Zn foil. When tested at a battery-level current density of 0.1 A g^−1^, the MCHS-coated Zn foil can circulate stably for 550 cycles, whereas the bare Zn foil suffered a large attenuation of capacity after only ~ 350 cycles. It is believed that the coat-layer strategy can strongly stabilize the depositing/stripping reaction of Zn foil in the conditions of both fast and slow charging/discharging process. Additionally, electrochemical impedance spectroscopy (EIS) measurement presented that the MCHS-coated Zn anodes have smaller charge transfer resistance (*R*_ct_, Fig. 2b) and faster ion diffusion rate than bare Zn foil, verifying the high-efficient Zn-ion depositing/stripping processes of MCHS-coated Zn anodes. Considering these results, the coat-layer strategy mentioned above may facilitate the splendid application of Zn foil in the ZHSCs field and then achieve better electrochemical performance.

#### Electrodeposited Nanostructure Zn Anode

By achieving a rough and relatively uniform surface, the electrodeposited Zn anode can greatly avoid the formation of Zn dendrite and realize higher electrochemical performance [93]. In addition, electrodeposited Zn materials have been widely used as the Zn anodes in ZIBs before being applied in ZHSCs [32, 94]. However, the strategy is pretty different between the ZIBs and ZHSCs, where the electrodeposited Zn anode for ZHSCs requires not only high energy density but also high power density.

As shown in the scanning electron microscope image (SEM, Fig. 2c) and illustration (Fig. 2d), Cha et al*.* proposed a well-designed nanostructure of hierarchically electrodeposited Zn anode (2D-Zn) for ZHSCs, where the hierarchical structure of Zn is constructed with the close-knit background Zn and dispersive 2D nanostructure Zn on the surface [53]. Moreover, the electrode depositing strategy is carefully investigated by the three kinds of the electrodeposited 2D-Zn anode. The high voltage of 1.8 V electrodepositing process can uniformly cover the Ni form with close-knit background Zn while the low voltage of 1.0 V electrodepositing process can cover the Ni form with dispersive 2D nanostructure Zn. Therefore, to achieve stable electron transport process and fast ionic diffusion rate simultaneously, the combined electrodepositing process of 1.8/1.0 V can manufacture a suitable hierarchical structure of electrodeposited Zn anode. Figure 2e depicts the EIS test results of three kinds of 2D-Zn anode. The hierarchical structure of 2D-Zn/1.8/1.0 electrodes presented the superb electrochemical performance of low *R*_*ct*_ and rapid ionic diffusion rate. Accordingly, two-step electrodepositing strategy can be a great method to synthesize the optimal nanostructure of Zn anode for ZHSCs, the as-assembled ZHSCs delivered an ultra-high energy density of 208 Wh kg^−1^ and a peak power density of 20,000 W kg^−1^. Meanwhile, remarkable cycling performance of 99% capacity retention after 10,000 cycles is achieved at 10 A g^−1^.

**Fig. 3:**
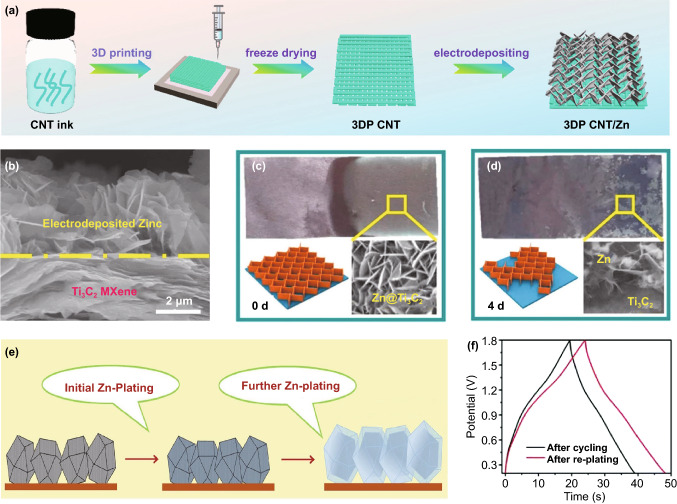
**a** Schematic illustration displaying the manufacturing process of 3DP-CNT/Zn anode. Reproduced with permission from Ref. [91]. Copyright 2021, American Chemical Society. **b** Cross-section SEM image of Zn@Ti_3_C_2_ anode. **c**, **d** Digital, SEM and schematic images of Zn@Ti_3_C_2_ anode after different time in PBS at 85 °C. Reproduced with permission Ref. [96] Copyright 2019, American Chemical Society. **e** Schematic illustration of the two-steps Zn plating on ZIF-8. Reproduced with permission from Ref. [90]. Copyright 2019, Elsevier. **f** Galvanostatic charge/discharge (GCD) curves of the devices after cycling and re-plating. Reproduced with permission from Ref. [97]. Copyright 2018, Royal Society of Chemistry

Since the formation of Zn dendrite can be effectively circumvented by utilizing electrodepositing strategy, the connection between electrodeposited Zn and substrate becomes very important, which directly relates to the electrochemical characteristics of Zn anode [95]. The substrate used for electrodepositing is a crucial part of achieving high-performance electrodeposited Zn anodes. Notably, the three-dimensional (3D) printing (3DP) technique is employed by Sun et al*.* to form carbon nanotubes (CNTs) micro-lattice substrate for Zn electrodepositing [91]. Figure 3a displays the illustration of woodpile structure of the 3DP-CNT substrate, the unique structure based on the 3DP technique equipped the Zn@3DP-CNT with outstanding conductivity. Typically, the 3DP technique is a novel strategy for fabricating substrate for Zn anode, and the stereostructure of Zn anode may further promote the development of the electrodepositing method.

To implement the combination of high energy density and high power density, the electrodeposited Zn anodes are usually designed as high-surface-area structures and thin flakes. Thus, the as-designed structure of electrodeposited Zn anode may lead to a faster degradation rate than Zn foil due to the accelerated corrosion interaction. As depicted in Fig. 3b, Zhi et al*.* vertically electrodeposited the Zn nanoflakes onto the Ti_3_C_2_ film, the thickness of the Zn nanoflakes is ~ 42 nm [96]. When the Zn@Ti_3_C_2_ electrode is soaked in the phosphate buffer saline solution (PBS, Fig. 3c, d) at 85 °C, part of Zn nanoflakes were dissolved into the PBS after 4 days, and finally eliminated as time prolonged to 7 days, which is much faster than the Zn foil (> 30 days). The degradable electrodepositing strategy makes the electrodeposited Zn anode and the ZHSCs more eco-friendly electrode and ESSs, respectively.

Porous structure of metal–organic framework (MOF) is also an excellent host for dendrite-free and fast diffusion Zn-ion storage. Wang et al*.* prepared MOF Zn-zeolitic imidazolate framework (ZIF-8) electrodes with a 3D cage structure via optimized heat treatment (500 °C) [90]. After the heat treatment, the Zn^2+^ turned into the evenly distributed Zn^0^ in porous ZIF-8–500, which can provide uniform nuclei for Zn depositing and high over-potential to reduce the H_2_O decomposition during the charging/discharging processes. As illustrated in Fig. 3e, the Zn electroplating process is divided into two steps, including the initial Zn plating and further Zn plating. Initial Zn plating normally proceeded in or on the ZIF-8-500 particle due to the uniform nuclei provide by the Zn^0^, and then further Zn plating occurred on the particle substrate and end with dendrite-free Zn@ZIF-8-500 anode. Subsequently, Zn@ZIF-8-500 anode is coupled with activated carbon cathode to form ZHSCs and delivered an energy density of 140.8 Wh kg^−1^ as well as splendid longevity up to 20,000 cycles. Similarly, Shen et al*.* employed annealed ZIF-8 as the substrate of dendrite-free Zn anode and Zapien et al*.* deposited the Zn onto the flexible carbon cloth (CC) [98, 99]. The combination of electrodeposited Zn and carbon materials enables the composite Zn anode outstanding properties for high-performance ZHSCs.

#### Recoverable Zn Anode

In the high-performance ZHSCs devices, the irreversible consumption of the Zn anode is unavoidable and the endurance of the battery-type Zn anode is not satisfied with the long-term supercapacitors [100]. Therefore, a suitable strategy that can refresh the Zn electrodes is necessary. Sun et al*.* reported recoverable Zn anodes for ZHSCs by applying an in-situ electroplating method [97]. After re-plating process, no clear morphology alteration is detected and the re-plating Zn nanosheets presented vertical orientation, which may provide a lower resistance pathway for electron transfer. As presented in Fig. 3f, the re-plating Zn nanosheets anodes exhibited a higher capacity (76 mF cm^−2^) than the after-cycling (before re-plating) Zn nanosheets anodes (60.9 mF cm^−2^). Furthermore, the re-plating Zn nanosheets anodes maintained the capacity for additional 1500 cycles. Therefore, this simple operation can efficiently retard the irreversible recession of Zn anode during charging/discharging processes. Notably, the re-plating routine was replenished with in-situ method, thus the device structure will stay intact. Therefore, electronic devices can be easily refreshed through specific charging processes, and the recycling projects of ZHSCs can expediently renew these devices and apply them to other low-demand electronic devices.

Considering the above three strategies for Zn anodes in ZHSCs, it is believed that the hinges of Zn anodes are circumvents of Zn dendrite and the improvement of electron transfer as well as ionic diffusion. Compared with bare Zn foil, the coating protection and well-designed electrodepositing strategies of the Zn electrode enable them more appropriate for irreversible and high-efficient Zn-ion depositing/stripping processes in ZHSCs. Also, the recoverable Zn anode method can promote the lossless realization of renewable ZHSCs. We believe that a well-designed Zn anode should be focused on both the energy-part and power-part. The excellent integration of energy-part and power-part may facilitate the promotion of energy density in ZHSCs.

### Cap Electrode

Cap electrode material is a vital part for both two electrode configurations of ZHSCs, which can equip the ZHSCs with good conductivity, fast charge/discharge rate and high electrochemical stability. Among the reported ZHSCs, two kinds of Cap electrodes are investigated, including the carbon electrode and pseudocapacitive electrode. Typically, carbon electrodes like high specific surface area (HSSA) carbon electrode, heteroatom-doped carbon electrode and graphene-based electrode can deliver an excellent power performance based on the fast adsorption/desorption reactions and considerable capacity for ZHSCs. Pseudocapacitive electrodes such as MXenes, polymer-carbon hybrid electrodes and transition metal compounds (TMCs) also present high capacity with some unique properties for ZHSCs. In the following section, we will review recent progress of the Cap electrodes and corresponding electrochemical performance in ZHSCs.

#### Carbon Electrode

The carbon electrodes with splendid properties of low cost, high chemical stability and good conductivity have been regarded as the optimal Cap electrode candidates for high-performance ZHSCs [31, 101, 102]. By proceeding the fast ion adsorption/desorption processes in the ZHSCs, the carbon electrodes can store some energy and further deliver considerable power performance. Nevertheless, due to the energy storage mechanism of EDLC, the normal carbon electrodes cannot offer sufficient energy because of the limited specific surface area (SSA). Thus, many strategies were proposed to promote the energy and power performance of carbon electrodes, such as (1) HSSA carbon electrode based on active carbon (AC), porous carbons (PCs), hollow carbon spheres (HCSs), flower-like carbons (FCs) and CNTs electrodes, (2) heteroatom-doped carbon electrodes. Additionally, graphene-based electrode is also a promising carbon electrode for ZHSCs. In this section, various recently reported carbon electrodes are expressly reviewed.

##### HSSA Carbon Electrode

**Fig. 4:**
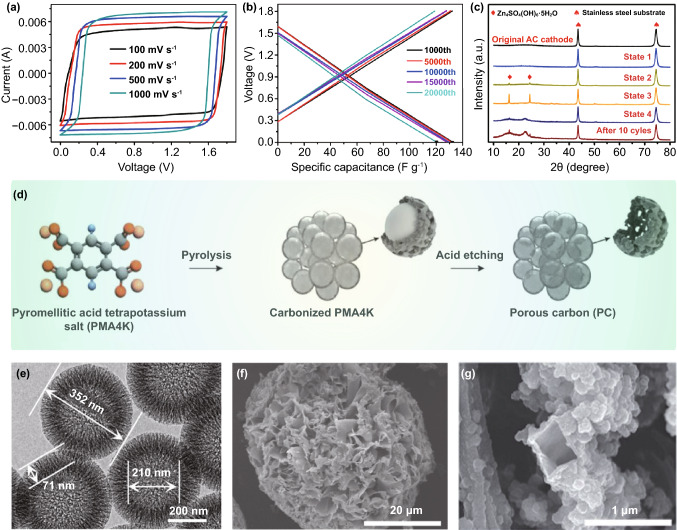
**a** CV curves and **b** GCD profiles of representative Zn//AC ZHSCs. Reproduced with permission from Ref. [101]. Copyright 2018, Elsevier. **c** XRD patterns of AC electrodes at different states. Reproduced with permission from Ref. [31]. Copyright 2018, Elsevier. **d** Schematic diagram illustrating the synthesis processes of PCs. Reproduced with permission from Ref. [108]. Copyright 2020, Wiley–VCH. **e** SEM image of HCSs. Reproduced with permission from Ref. [92]. Copyright 2020, Elsevier. **f** SEM image of FCs. Reproduced with permission from Ref. [109]. Copyright 2021, Elsevier. **g** SEM image of CNTs. Reproduced with permission from Ref. [110]. Copyright 2019, Royal Society of Chemistry

As mentioned above, the carbon electrode is the representative cathode for Zn//Cap ZHSCs. Due to the energy storage mechanism of adsorption/desorption reaction, the capacity is highly connected with the SSA of electrode materials, thus, common strategies focus on implementing the HSSA carbon cathode [103–106]. The conventional AC materials were reported by Tang et al*.* and the corresponding Zn//AC ZHSCs were fully investigated [101]. To some extent, the AC is a microporous carbon material with HSSA. As shown in Fig. 4a, the rectangular shapes of CV curves demonstrated that the Zn//AC ZHSCs have typical Cap energy storage behaviors, whereas the energy density (52.7 Wh kg^−1^) of the ZHSCs is much more than normal supercapacitors [107]. After 20,000 cycles of charging/discharging processes (Fig. 4b), the Zn//AC ZHSCs maintained stable electrochemical behavior, and the capacity had no evident decline (remained 91% of initial capacity). Although the ultra-stable property of commercial AC materials provides the ZHSCs with ultra-long longevity, the energy density of as-prepared ZHSCs is not high enough for practical applications. Thus, Kang et al*.* optimized the electrochemical performance with AC powders and the Zn//AC powders ZHSCs offered a higher capacity of 84 Wh kg^−1^ [31]. Gratifyingly, the Zn//AC ZHSCs exhibited a splendid cycling performance (91% after 10,000 cycles) at a very low current density of 1 A g^−1^, which is almost the battery-level cyclic condition but the longevity is ten times longer than batteries. Accompanying with the normal adsorption/desorption reaction, the precipitation and dissolution processes of by-products (Zn_4_SO_4_(OH)_6_·5H_2_O) is detected by the XRD spectra (Fig. 4c) of AC powders [31]. In addition, more by-products like ZnSO_3_·2.5(H_2_O) were demonstrated in various ZHSCs during the charging/discharging processes [34, 77, 103]. The effects and particular mechanisms of the by-products will be carefully discussed in Sect. 3.

Apart from the commercial AC, many PCs electrode materials can be fabricated through various methods, like template method [111–113] and pyrolysis method [108, 114–116]. As for the pyrolysis methods, the precursor is a significant part of synthesizing high-quality PC electrode materials. Lu et al*.* reported molten salt-assisted synthesis of pitch-derived PCs cathodes for Zn//Cap ZHSCs while Guan et al*.* proposed bamboo-derived PCs cathodes with superb stability when employed in ZHSCs (96% capacity retention after 90,000 cycles) [114, 115]. Moreover, Xu et al*.* and Shen et al*.* reported a MOF route for fabricating PCs electrode materials, the ZIF-8-derived PCs presented an splendid performance of 225 mAh g^−1^ at 0.1 A g^−1^ and remained 135.5 mAh g^−1^ at 1 A g^−1^ [98, 116]. As depicted in Fig. 4d, Alshareef et al*.* reported a two-step synthesis method including pyrolysis at 800 °C and acid etching for manufacturing PC-800 cathode [108]. After then, the PC-800 cathode showed additional pseudocapacitance which are originated from the variation in oxidation states of oxygen functional groups. The as-assembled Zn//PC-800 ZHSCs reached a high capacity of 179.8 mAh g^−1^ and surprising capacity retention of 99.2% after 30,000 cycles.

HCSs, which possess hollow structures and micro- or nano-shells, are regarded as a promising HSSA carbon cathode as well. The hollow structure enables the HCSs cathode great specialties of high surface-to-volume ratio and substantial accessible sites. Chen et al*.* presented the HCSs cathode through the calcination of co-polymer and applied a surfactant as the soft template [99]. After that, the HCSs cathode matched with a flexible Zn@CC anode to assemble Zn@CC//HCSs ZHSCs. The HCSs have a uniform size of ~ 100 nm external diameter and ~ 50 nm inner diameter, and the Brunauer–Emmett–Teller (BET) surface area of HCSs was measured to be 819.5 m^2^ g^−1^. Benefiting from the HSSA property and hollow structure of HCSs, the ZHSCs achieved energy density of 59.7 Wh kg^−1^ in a low concentration of 0.08 M ZnSO_4_ polyacrylamide (PAM) hydrogel electrolyte and ultra-stable cycling performance (98% of initial capacity) after 15,000 cycles. Due to the low concentration and solid-state property of the applied electrolyte, the energy density of the flexible Zn@CC//HCSs ZHSCs is limited compared to aqueous ZHSCs. To further investigate the performance of HCSs cathode in the aqueous ZHSCs, Liu et al*.* demonstrated MCHSs cathodes with ultra-high BET surface area of 1275 m^2^ g^−1^ [92]. As shown in Fig. 4e, the external and inner diameters of MCHSs are 352 and 210 nm, respectively. With the increasing mass loading of electrodes, the HSSA carbon cathode may suffer from worsening ion and charge transport characteristics in thicker electrodes. However, the interconnected porous of MCHSs can provide a short ion and charge transport path as well as good penetration, leading to its excellent electrochemical performance of ZHSCs even under the condition of high mass loadings. Hence, the as-assembled ZHSCs can deliver a remarkable energy density of 129.3 Wh kg^−1^ and 100% capacity retention after 1000 cycles at 0.1 A g^−1^. When increasing the current density and mass loading, the MCHS-based ZHSCs only showed a small capacity degradation.

Recently, a new HSSA carbon cathode of FCs was applied by Hu et al*.* as the carbon cathode for Zn//Cap ZHSCs [109, 117]. The hierarchical 3D FCs are formed by the spheroidizing growth processes of carbon and low-dimensional carbon nanosheets, which possess large surface area, large surface-to-volume ratios, interconnected conductive network, nanoscale architecture and abundant accessible active sites. The SEM shown in Fig. 4f exhibited that the diameter of the 3D FCs is ~ 40 μm and the size of the carbon nanosheets is ~ 2 × 2 μm^2^. Based on the unique structure of 3D FCs, the Zn//FC ZHSCs offered 119.7 Wh kg^−1^ at 890 W kg^−1^ and splendid cycling performance of 92% capacity retention after 20,000 cycles. In addition, 1D CNTs displayed in Fig. 4g can also be used as HSSA carbon electrodes for ZHSCs depend on their special property of decent electrical conductivity, splendid mechanical property, high chemical stability and uniform distribution of pore size [118, 119]. Although the reported CNTs electrodes possess a low SSA of 200–300 m^2^ g^−1^ compared with PCs and HCSs electrodes, the CNT-based carbon electrode can offer incredible capacity for ZHSCs [110, 120, 121]. Han et al. applied B, N co-doped porous carbon microtube (BN-CMTs) as the cathode of ZHSCs and ultimately achieve an ultra-high capacity of 472.6 Wh kg^−1^ [110]. Moreover, research showed that the electrochemical characteristics of CNTs can be highly improved by many activation methods depending on their intrinsic properties [119, 122]. Based on the excellent mechanical property, Chen et al*.* applied CNTs as the flexible substrate for both cathode and anode materials, assembling a highly flexible fiber-type ZHSCs [120]. The as-prepared ZHSCs can keep similar CV curves under bent and twisted conditions, delivered an outstanding specific capacity of 104.5 F cm^−3^ and steady cycling performance of 98.5% capacity retention after 10,000 cycles.

After reviewing the reported HSSA carbon electrode of ZHSCs, one phenomenon can be discovered that the relationship between capacity and surface area is not linear. Two HSSA carbon electrodes with similar BET surface area and configuration of ZHSCs may offer disparate energy density. From our point of view, it can be ascribed to overlapping effects and electronic kinetics. Although the HSSA carbon electrodes are achieved by various nanostructures, the effective solution–surface contact area varies from structure to structure. Sometimes, the inside carbon materials can be shielded by external carbon materials, thus leading to invalid surface area for EDLC. Similarly, the electronic kinetics are also affected by different surface morphology and mass loading of carbon electrodes. Hence, the obsession for extremely high SSA carbon electrodes is inefficient and counter-productive. On the contrary, prominent nanostructure and modified component of carbon electrode with suitable SSA may be the way for realizing higher performance ZHSCs.

##### Heteroatom-Doped Carbon Electrode

Particularly, heteroatom doping is an efficient way to modify the physical and chemical properties of carbon electrodes [123–125]. By replacing part of C atoms with other elements like B, N and P, the strategy can not only optimize the synthesis process of heteroatom-doped carbon electrodes but also further improve the electrochemical performance of the as-prepared ZHSCs [126]. For one, heteroatom doping can improve the electrical conductivity, create more electrolyte accessible active sites and alleviate agglomeration of carbon electrodes [127]. For another, heteroatom doping can accelerate the permeation and diffusion character of electrolytes, thus further promote the wettability of carbon electrodes [128, 129].

**Fig. 5:**
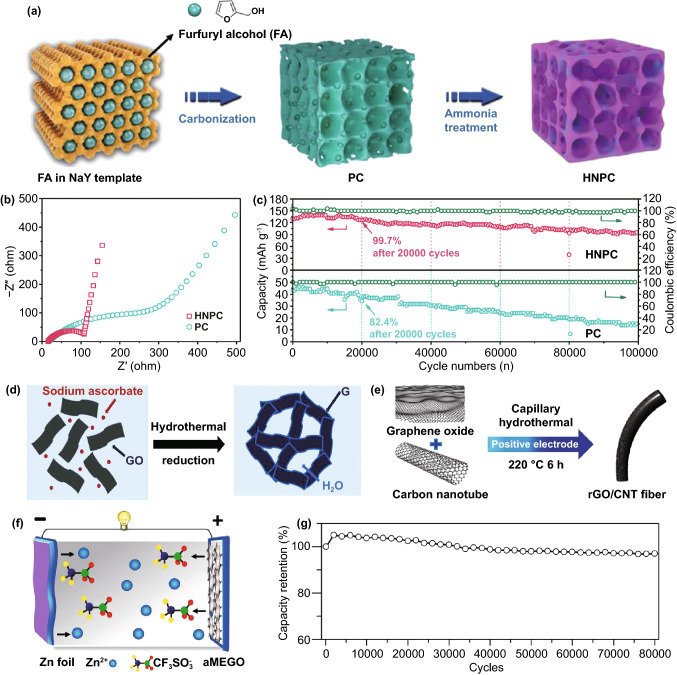
**a** Schematic illustration of the synthesis route of the HNPC. **b** Nyquist plots and **c** cycling performance at 16.7 A g^−1^ of the Zn//PC and Zn//HNPC ZHSCs. Reproduced with permission from Ref. [85]. Copyright 2019, Wiley–VCH. **d** Schematic of the preparation process of 3D graphene by hydrothermal reduction. Reproduced with permission from Ref. [131]. Copyright 2018, Royal Society of Chemistry. **e** Schematic illustration of the synthesis of rGO/CNT. Reproduced with permission from Ref. [120]. Copyright 2019, Wiley–VCH. **f** Schematic illustration of discharging process and **g** cycling performance of the Zn//aMEGO ZHSCs. Reproduced with permission from Ref. [82]. Copyright 2019, Wiley–VCH

Accordingly, Lu group reported that the N doping is expected to favor the chemical absorption of Zn-ions and tremendously altered the properties like conductivity, surface wettability, electrochemically active surface area as well as ion/electron transportation of original PCs [85]. The synthesis illustration of HNPC is shown in Fig. 5a. Particularly, the HNPC-based ZHSCs can offer an ultra-high capacity of 177.8 mAh g^−1^, which is almost three times higher than non-N-doped PC and higher than most carbon-based electrodes. Through the EIS tests shown in Fig. 5b, the fantastic promotion of N-doping strategy in decreasing charge transfer resistance and enhancing the diffusion rate was demonstrated compared with original PCs electrodes. Furthermore, the strategy of heteroatoms doping can even boost the stability of HNPC electrodes. As depicted in Fig. 5c, the HNPC-based ZHSCs maintained 99.7% capacity retention after 20,000 cycles while the PC-based ZHSCs displayed a lower capacity retention of 82.4%. Liu group also proposed an N-doped hierarchical porous carbon (N-HPC) which is derived from commercial chitosan via simultaneous carbonization and activation [130]. Gratifyingly, a remarkable energy density of 191 Wh kg^−1^ was achieved by N-HPC-based ZHSCs.

Apart from N atoms, P dopant has been reported in the carbon electrodes of ZHSCs as well, which possesses a lower electronegativity of 2.19 than N atoms [127]. Chen et al*.* prepared P-doped honeycomb-like porous carbon (PHCA) electrodes by replacing the C atoms in carbon skeletons with P dopant [112]. Subsequently, the P dopants in carbon skeletons lead a chemical bonds transformation from C–OH (C–C–OH) to P–C–OH, which results in a stronger polarization of the hydroxyl group and lower energy barrier (from 0.13 to 0.04 eV). The stronger polarization of the hydroxyl group and lower energy barrier further enable a more attainable reaction during charging/discharging processes. Based on the PHCA electrodes, the Zn//PHCA ZHSCs presented a peak energy density of 129.3 Wh kg^−1^ at a power density of 1000 W kg^−1^.

Besides the single heteroatom-doping strategy, co-doping strategy will further promote excellent performances of carbon electrodes based on the synergistic effect of diverse heteroatoms. N, O co-doped hierarchical porous carbon (NO-HPC) was employed as the cathode in ZHSCs by Zhao group, where the ZIF-8 is applied as the template and C source [132]. Although the SSA of the carbon electrode is relatively low (197.45 m^2^ g^−1^), the Zn//NO-HPC ZHSCs exhibited splendid energy density of 110 Wh kg^−1^ and lossless capacity after 10,000 cycles. In addition, P, B co-doped AC (PB-AC) is reported by An et al*.* and the Zn//PB-AC ZHSCs delivered a high energy density of 169.4 Wh kg^−1^ and superb longevity of 30,000 cycles [133]. Furthermore, the second-highest energy density (472.6 Wh kg^−1^) of reported ZHSCs is based on the B, N co-doped BN-CMTs electrodes, which is even higher than most of ZIBs and LIBs [110]. However, the SSA of the BN-CMTs (101.24 m^2^ g^−1^) is much lower than most HSSA carbon electrodes mentioned above. It is believed that the B, N co-doping strategy facilitates the faradaic reactions of pseudocapacitance on the electrode surface by changing the electronic structure and density state of carbon, thus realizing prominent energy density even under a relatively low SSA. In short, heteroatom doping is an effective and promising strategy for enhancing the electrochemical performance of carbon electrodes. However, the deep working mechanisms of carbon electrodes heteroatom doping in ZHSCs are still in need. A better understanding of heteroatom doping in ZHSCs is highly significant, which leads to reasonable dopants and reproducible high performance of ZHSCs.

##### Graphene-Based Electrode

In addition to normal active carbon materials, graphene is the most common material used in symmetric supercapacitors [134]. As a novel 2D material with excellent properties such as decent intrinsic electric conductivity in-plane, high theoretical SSA of 2630 m^2^ g^−1^, outstanding mechanical strength and chemical stability, graphene and its derivatives have been widely applied in many ESSs [134–136]. The intrinsic capacitance of monolayer graphene can reach ~ 21 μF cm^−2^ according to its theoretical SSA [137]. Also, graphene-based materials are characterized by ultra-low RC constants of less than 200 μs, while the number is about 1 s for typical carbon materials [138]. Hence, it is generally believed that graphene-based materials is a promising candidate for high-performance ZHSCs.

As shown in Fig. 5d, Han et al*.* fabricated graphene-based electrodes for ZHSCs, where the reduced graphene oxide electrodes (G) are derived from the graphene oxide (GO) via hydrothermal reduction [131]. After further modified the G electrodes with polyaniline (PANI) by novel in-situ polymerization, the highest energy density (205 Wh kg^−1^) in graphene-based ZHSCs was achieved. However, the G electrodes without further polymerization showed a better cycling performance than G@PANI electrodes, indicating the trade-off between energy density and cycling stability. As a novel material, graphene was widely employed for the modification of other carbon electrodes in ZHSCs. For instance, Wang et al*.* and Xu et al*.* improved the cycling performance of ZHSCs by introducing graphene materials into the electrodes [139, 140]. As illustrated in Fig. 5e, Chen group demonstrated a flexible fiber-shaped reduced graphene oxide (rGO) on CNT substrate by capillary hydrothermal method [120]. Moreover, the rGO//CNT cathode was coupled with the electrodeposited Zn//graphene fiber anode to form ZHSCs. Benefiting from the both graphene-based electrodes, the flexible ZHSCs device delivered a splendid energy density of 48.5 mWh cm^−3^ and decent longevity of 10,000 cycles with merely 1.7% capacity decay.

Furthermore, the ultra-high stability of graphene-based electrodes in ZHSCs was confirmed by various investigations. For one, Wang et al*.* developed a porous 3D MXenes-rGO aerogel cathode for ZHSCs with an ultra-long cycling lifetime (95% capacity retention after 75,000 cycles) [139]. The introduced MXenes (Ti_3_C_2_T_*x*_) in the composite electrodes can provide an additional pseudocapacitive reaction of Zn-ion insertion/desertion, resulting in an improvement of ZHSCs capacity. For another, Zhang group fabricated PC electrodes derived from chemical activated microwave exfoliated graphene oxide (aMEGO) via three-step process of microwave treatment, KOH activation and washing-drying-annealing [134]. As depicted in Fig. 5f, the structure of Zn//aMEGO ZHSCs is illustrated [82]. By applying 3.0 M Zn(CF_3_SO_3_)_2_ and Zn foil as the electrolyte and anode, respectively, the Zn//aMEGO ZHSCs are assembled and provided a battery-level energy density of 106.3 Wh kg^−1^ and a capacitor-level power density of 31,400 W kg^−1^. Furthermore, it can achieve incredible stability that remained 93% of initial capacity after 80,000 charging/discharging cycles at 8 A g^−1^ (Fig. 5g). These two cases are tested in different electrolytes where the former is 2.0 M ZnSO_4_ and the latter is 3.0 M Zn(CF_3_SO_3_)_2_. Hence, the general adaptability and stability of graphene-based materials in ZHSCs are prominent, and the graphene-based electrodes are proved to be a great choice for high-performance ZHSCs.

#### Pseudocapacitive Electrode

Although featured with HSSA, special morphologies and splendid electrical conductivity, the energy densities of most pure carbon electrodes are still far from practical applications due to the limited energy storage mechanism of EDLC based on simple physical adsorption/desorption processes. By contrast, pseudocapacitive materials with the co-existence of reversible faradic reactions and EDLC are promising candidates for large capacity of ZHSCs [141, 142]. As we mentioned above, the heteroatom-doped carbon material is a typical pseudocapacitive material for ZHSCs, and the pseudocapacitive mechanisms have been already discussed. Except the heteroatom-doped carbon materials, many carbon-based composite electrodes combined with carbon materials and pseudocapacitive materials can present noteworthy pseudocapacitance, however, have not been applied for ZHSCs [143]. As the important component of pseudocapacitive materials, we believed that more and more carbon-based pseudocapacitive materials with composite energy storage mechanisms will be investigated in the system of ZHSCs, and then lead to high-performance ZHSCs. In the following section, we are going to discuss some reported works of pseudocapacitive electrodes such as MXenes, polymer-carbon hybrid materials and TMCs (e.g., RuO_2_, TiN), and analyze the corresponding electrochemical performance of as-prepared ZHSCs.

##### MXenes

MXenes, the prominent family of 2D transition metal carbide and nitrides, are widely investigated and employed in many ESSs like LIBs and supercapacitors [26, 144–147]. It is generally demonstrated that the MXenes electrodes can be hosts for a range of cations such as Na^+^, K^+^, NH_4_^+^, Mg^2+^ and Zn^2+^ [148, 149]. Additionally, the ions penetrations only occur between the MXene sheets because the chemical bonds between M and X are unbreakable, thus occupying electrochemically active sites on the MXenes surfaces and realizing energy storage [150, 151]. Although the CV curves of MXenes electrodes are rectangular shapes without pronounced redox peaks, the energy storage mechanism of MXenes in acid is prominently proved to be pseudocapacitive [152]. As a promising candidate of pseudocapacitive cathode for ZHSCs, MXenes have attracted numerous attentions. Hence, some currently reported researches of MXenes on ZHSCs will be reviewed as follows.

Among the family of MXenes, the Ti_3_C_2_T_*x*_ is the most studied MXene for capacitors, which can achieve outstanding capacities of 300–400 F cm^−3^, surpassing most all-carbon-based super capacitors [148, 152]. Accordingly, Zhi group applied Ti_3_C_2_ electrode as the cathode of ZHSCs and further fabricated a flexible ZHSCs with the wholly degradable property [96]. The capacity of Zn@Ti_3_C_2_//Ti_3_C_2_ ZHSCs is enhanced to 330 F cm^−3^, which can be ascribed to the abundant pseudocapacitive reactions. Notably, the Zn-Ti_3_C_2_ ZHSCs can totally degrade within 7 days (Fig. 3c, d), which drastically exceeding the current supercapacitors of ~ 120 days and batteries of ~ 20 days. However, the decent degradable performance is realized at the cost of cycling longevity due to the relatively low thickness of Zn-Ti_3_C_2_. Therefore, the Zn@Ti_3_C_2_//Ti_3_C_2_ ZHSCs can only maintain capacity retention of 82.5% after 1000 cycles. To promote the longevity of Ti_3_C_2_-based ZHSCs, many efforts have been devoted. As we reviewed above, graphene-based ZHSCs showed superb cycling performance based on the extraordinary chemical stability. Therefore, Wang et al*.* prepared an MXenes-rGO electrode with the both excellent performance of MXenes and rGO [139]. The Zn//MXenes-rGO ZHSCs presented a prolonged cycling lifetime of 75,000 cycles and outstanding capacity retention of 95%. Furthermore, an in-situ annealing strategy is employed by Shen group to optimize the cycling performance of Ti_3_C_2_T_*x*_-based ZHSCs [153]. Before the annealing, the Ti_3_C_2_T_*x*_-based ZHSCs remained only ~ 54.7% of initial capacity after 5000 cycles. However, after annealed at 300 °C for 30 min (Ar atmosphere), the Ti_3_C_2_T_*x*_ electrodes presented ultra-long lifetime and outstanding stability (~ 80% after 50,000 cycles), which can be attributed to two reasons: (1) surface treatment of oxygen-containing functional group, (2) the generation of micropores in the Ti_3_C_2_T_*x*_ structure.

**Fig. 6:**
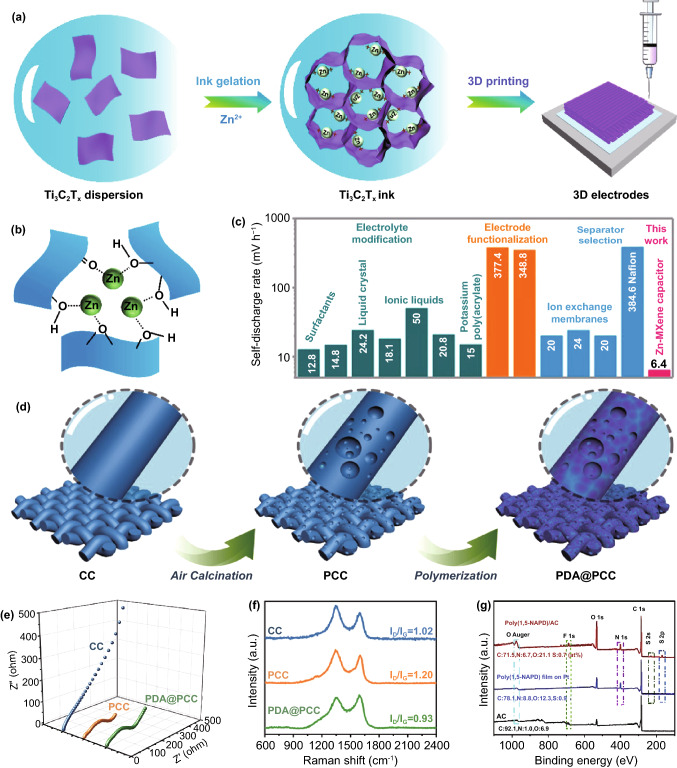
**a** Illustration of MXenes ink formulation and the 3DP process. **b** Schematic illustration of the Zn-ion gelation process targeting the preparation of MXenes ink. Reproduced with permission from Ref. [91]. Copyright 2021, American Chemical Society. **c** Comparison of self-discharge rate between the Zn//MXenes capacitor and previously reported anti-discharge supercapacitors. Reproduced with permission from Ref. [96]. Copyright 2019, American Chemical Society. **d** Preparation process of the PDA@PCC electrode. **e** Nyquist plots and **f** Raman spectra of CC, PCC and PDA@PCC electrodes. Reproduced with permission from Ref. [154]. Copyright 2020, American Chemical Society. **g** XPS spectra of poly(1,5-NAPD)/AC cathode, poly(1,5-NAPD) film on Pt, and AC coating. Reproduced with permission from Ref. [77]. Copyright 2020, Elsevier

As shown in Fig. 6a, Sun group reported a novel synthesis process of Ti_3_C_2_T_*x*_ electrodes by 3DP technology [91]. The free-design property of 3DP technology enables more possibilities of Ti_3_C_2_T_*x*_ electrodes including structure, thickness, size, etc. Significantly, the Ti_3_C_2_T_*x*_ ink is the key part for 3DP technology, as depicted in Fig. 6b, the rheological behavior of Ti_3_C_2_T_*x*_ ink is altered via introducing the Zn-ion gelation. The electrostatic attraction between Zn^2+^ and negative charges on Ti_3_C_2_T_*x*_ further decrease the repulsion of adjacent Ti_3_C_2_T_*x*_ sheets. When assembled into the ZHSCs, the 3DP Ti_3_C_2_T_*x*_-based ZHSCs provide a splendid capacity of 1006.4 mF cm^−2^ at 0.1 A g^−1^ and rate capability (remain 71% when the current density up to 10 A g^−1^). Moreover, laser writing was applied by Li et al. to prepare Ti_3_C_2_T_*x*_ electrodes, which is facile and matched with micro ZHSCs technology [153].

Furthermore, it is well known that self-discharge behavior is common and fatal for supercapacitors [155]. Lu et al*.* investigated the self-discharge phenomenon of BN-LDC//Zn ZHSC devices, where the devices can retain a high voltage of 1.4 V after 24 h self-discharge compared to the initial voltage of 1.8 V, offering a fast self-discharge rate of 16.7 mV h^−1^ [83]. However, as shown in Fig. 6c, Zhi et al*.* prepared Zn@Ti_3_C_2_//Ti_3_C_2_ ZHSCs with lowest self-discharge rate of 6.4 mV h^−1^ (from 1.35 to 0.89 V after 72 h) among various supercapacitors which possess specific anti-self-discharge modification toward the electrode, electrolyte and separator [96, 156–159]. The key to this excellent behavior lies at the tight fixation between reduced Zn atoms and the anodic surface of MXenes electrodes under open-circuit state, thus, the Zn atoms will not transfer to cathodic area, leading to the remarkable anti-self-discharge property of MXenes. Although numerous advantages MXenes have shown, the operating voltage window of MXenes-based ZHSCs is quite narrow (0–1.4 V), which is limited for practical applications. Therefore, more efforts should be made to promote the higher operating voltage and better cycling performance of MXenes in the future.

##### Polymer-Carbon Hybrid Electrode

Redox-active conducting polymers are also decent pseudocapacitive materials featured with fast and reversible redox reactions, thus the conducting polymer-based supercapacitors are able to provide higher capacity than those EDLC-only Cap materials. However, conducting polymer electrodes also cannot satisfy the longevity demand of high-performance supercapacitors due to the poor stability, active material loss and over-oxidative degradation during charging/discharging processes [160]. Hence, lots of studies try to solve the problem with the polymer-carbon hybrid electrode, where the ultra-stable carbon materials act as the substrate and the conducting polymer materials act as the active mass loadings. The excellent synergetic effect enables the polymer-carbon hybrid materials to become suitable electrodes for ZHSCs.

Gratifyingly, many high-performance ZHSCs based on polymer-carbon hybrid electrodes have been reported recently, including different carbon substrates like CC, AC and rGO [77, 140, 154]. Han et al*.* employed PANI and rGO as the component of polymer-carbon hybrid electrodes and applied aniline solution on the surface of graphene hydrogel for the in-situ polymerization method [131]. Surprisingly, the PANI@rGO-based ZHSCs presented a capacity of 154 mAh g^−1^, which is five times higher than individual rGO electrodes. However, the PANI@rGO-based ZHSCs displayed an inferior capacity retention of 80.5% after 6000 charging/discharging cycles. Similarly, Hu group modified the rGO materials with another conducting polymer of p-phenylenediamine (PPD), where the PPD is inserted into the interlayer of rGO, resulting in bigger interlayer spacing and thus expose more ion accessible sites [140]. The PPD@rGO hybrid electrode is fabricated by facial thermal evaporation and hydrothermal process. As a result, PPD@rGO-based ZHSCs presented an incredible capacity of 3012.5 mF cm^−2^ at a current density of 1 mA cm^−2^, which is 11.2 times higher than the rGO-based ZHSCs provided. The excellent cycling performance of 100% capacity retention after 4000 cycles was realized by PPD@rGO-based ZHSCs as well. Furthermore, Liu et al*.* prepared a poly(1,5-naphthalenediamine, NAPD)/AC hybrid electrode via a direct electrodepositing process [77]. Interestingly, the helix-shaped poly(1,5-NAPD) chains tend to be bundled up during the depositing process and ended as salient nanorods. By modifying the morphology of normal AC electrodes, ions can be efficiently inserted/extracted from the molecular chains, and consequently, the poly(1,5-NAPD)/AC-based ZHSCs presented a remarkable energy density of 195 Wh kg^−1^ and 91% capacity retention after 10,000 cycles. Hence, there is no doubt that the polymer-carbon hybrid electrode can tremendously improve the capacity of carbon materials and relatively stabilize the unstable structure of conducting polymer.

To further investigate the inner mechanism of polymer–carbon hybrid electrode, Huang et al*.* reported a polydopamine-coated porous carbon cloth (PDA@PCC) electrode and the preparation process of PDA@PCC electrode is illustrated in Fig. 6d [154]. Figure 6e presents the Nyquist plots of three different electrodes of CC, PCC and PDA@PCC electrodes. Typically, PDA@PCC and PCC showed a shorter tail than CC, which represented a better ion diffusion ability based on the hydrophilic property and pore structures. However, due to the defects of PDA polymerization, PDA@PCC exhibited a relatively larger semicircle in the high-frequency region. The Raman spectra of diverse electrodes are studied in Fig. 6f, the intensity ratio (*I*_*D*_/*I*_*G*_) of D band and G band generally indicates the disorder degree of carbon-based materials. The PDA@PCC hybrid electrodes possess the lowest ratio of 0.93, which is beneficial for achieving stable electrochemical performance. It is believed that the introduction of PDA can greatly restore the disorder degree from 1.20 to 0.93 and retain the porosity of PCC during the synthesis processes of PDA@PCC. Hence, with the lowest disorder degree, PDA@PCC-based ZHSCs delivered a great capacity retention of 100% after 10,000 cycles. Moreover, Fig. 6g shows the XPS spectra of poly(1,5-NAPD)/AC, poly(1,5-NAPD)/Pt and bare AC samples. It can be proved that the synergetic effect of polymer–carbon hybrid electrode is not simple superposition but proportional integration according to the N 1 s spectra of each electrode. Simultaneously, the weak S and F signals were reported as the binder between the poly(1,5-NAPD) and AC during the electrodepositing process.

##### Transition Metal Compounds (TMCs)

TMCs electrode materials such as transition metal oxides (TMOs), nitrides (TMNs), hydroxides and sulfides have been widely investigated in the metal-ion batteries and capacitors on account of their outstanding characteristics like multiple metal valences, high theoretical capacitance, abundant natural source and diversiform synthetic methods [161, 162]. Hydrous RuO_2_·H_2_O, as the first demonstrated pseudocapacitive materials, have been used as the advanced Cap electrode materials in many ESSs [163, 164]. As a result, Dong et al*.* employed amorphous RuO_2_·H_2_O as the cathodes of Zn//Cap ZHSCs based on a fast and safe Zn-ion pseudocapacitive storage mechanism [165]. The electrochemical performance of anhydrous RuO_2_ and hydrous RuO_2_·H_2_O were compared in the system of ZHSCs. Although the anhydrous RuO_2_ possess higher electrical conductivity than hydrous RuO_2_·H_2_O, the bound H_2_O in RuO_2_ can facilitate the ions diffusion and thus lead to a better capacity than anhydrous RuO_2_. According to Dunn’s method, 79.0–96.4% capacitance of RuO_2_·H_2_O cathodes originates from the surface-controlled capacitive process. The capacitance is ascribed to the co-existence of EDLC and redox pseudocapacitance, and the majority part of the capacitance is devoted by the pseudocapacitance since the SSA of RuO_2_·H_2_O electrodes is only 57 m^2^ g^−1^. Benefiting from the high conductivity (> 100 S cm^−1^) and abundant surface redox reactions of hydrous RuO_2_·H_2_O, the assembled Zn//RuO_2_·H_2_O ZHSCs enabled ultra-fast Zn-ion storage speed and presented splendid energy density of 119 Wh kg^−1^ with a voltage window of 0.4–1.6 V. Particularly, it still kept a high energy density of 82 Wh kg^−1^ under an ultra-high power output of 16,740 W kg^−1^. It is amazing to reach such balanced electrochemical performance while the reported LIBs and ZIBs can only deliver a power density of 1–10 kW kg^−1^. Moreover, the rate performance of the Zn//RuO_2_·H_2_O ZHSCs is superior than many TMCs and the ZHSCs also presented an outstanding capacity retention of 87.5% after 10,000 cycles at a current density of 20 A g^−1^.

Titanium nitride (TiN) with superb electrical conductivity (27 μΩ cm^−1^) and chemical/electrochemical stability have also been considered as a promising pseudocapacitive electrode material for energy storage [166–168]. Zhi group employed the TiN electrode as the cathode materials for Zn//Cap ZHSCs, and coupled with Zn metal anode to form Zn//TiN ZHSCs [169]. Although TiN is a pseudocapacitive material, no evident redox peaks of TiN electrodes are detected in the CV curves during charging/discharging process, indicating the predominant Cap behaviors of the TiN cathode materials in the system of ZHSCs. Owing to the excellent conductivity and splendid stability of TiN, the Zn//TiN ZHSCs delivered an ultra-high capacity of 489.8 F g^−1^ at 0.2 A g^−1^ in the ZnSO_4_ electrolyte. In addition, the self-discharge rate of TiN-based ZHSCs is extremely low due to the high stability of TiN-SO_4_ structure after adsorption process, which remained 83.9% of initial capacity after 500 h resting time.

Herein, it is believed that the TMCs materials with diverse properties and Zn-ion storage mechanisms are competitive pseudocapacitive electrodes for high-performance ZHSCs. However, we need to draw a distinction between the pseudocapacitive TMCs and the battery-type TMCs like manganese oxides and vanadium oxides, as well as other reported ZBC. Compared with the Cap TMCs electrodes, the redox process of battery-type TMCs is responsible for the phase transition of electrode materials [142]. Additionally, the potential of the battery-type electrodes will remain approximately constant, presenting a distinct charging/discharging platform in the GCD curves, which agrees with the phase rule and follows the Nernst equation. Correspondingly, a couple of well-defined redox peaks can be detected in the CV curves. Hence, the pseudocapacitive TMCs electrodes performed as the Cap cathode in the Zn//Cap ZHSCs while the battery-type TMOs acted as the ZBC in the Cap//ZBC ZHSCs.

In summary, Cap electrode materials including HSSA carbon electrodes, heteroatom-doped carbon electrodes, graphene-based electrodes and pseudocapacitive electrodes (MXenes, polymer-carbon hybrid electrodes and TMCs) are the most investigated materials among all candidate electrodes for ZHSCs. HSSA carbon electrodes can provide sufficient EDLC and power density for ZHSCs, however, the EDLC is not enough to offer balanced energy density which should match with battery-type electrodes in ZHSCs. Graphene-based electrodes possess excellent properties of ultra-high intrinsic capacitance (~ 21 μF cm^−2^), high theoretical SSA of 2630 m^2^ g^−1^, ultra-low RC constants (< 200 μs) and high chemical stability. Therefore, graphene-based ZHSCs present better longevity than other ZHSCs. Heteroatom-doped carbon electrodes and polymer–carbon hybrid electrodes are both based on the fundamental carbon electrodes, equipping the carbon electrodes with abundant chemical pseudocapacitive reactions, which can effectively enhance the energy density of normal carbon electrodes. As for polymer–carbon hybrid electrodes, although the energy density of carbon electrodes is promoted, the conducting polymer materials show poor cycling performance on account of the poor stability, active material loss and over-oxidative degradation. MXenes show balanced electrochemical performance but suffer from the low operating voltage window and short longevity. Notably, owing to the superb electrical conductivity and considerable pseudocapacitance, TMC-based ZHSCs achieve large capacity and fast charging/discharging rate. Therefore, among these reported Cap electrodes, graphene-based electrodes, heteroatom-doped carbon electrodes and TMCs electrodes are considered as the most promising Cap electrodes for the practical application of ZHSCs, and it is believed that more and more excellent Cap electrodes can be discovered as the fast development of ZHSCs. When designing an appropriate Cap electrode for ZHSCs with perfect performance, it is significant to keep the balance between power density, energy density and stability, which can be further attributed to the balance between EDLC and chemical pseudocapacitive reactions.

As overviewed above and summarized in Table 1, the Zn//Cap ZHSCs with different kinds of electrode materials have been expatiated and further concluded. More importantly, the intricate and unique storage mechanisms of Cap electrodes are carefully summarized as several parts, where the adsorption/desorption processes based on the EDLC are always effective while the redox reaction or insertion/desertion processes may exist depending on the diverse strategy of Cap cathodes. Based on the well-designed Zn anodes and various kinds of Cap electrodes, reported Zn//Cap ZHSCs showed ultra-long cycling lifetime and excellent integration of high energy density and power density. The Zn//Cap ZHSCs can reach the approximate energy density of LIBs but higher power density, making the ZHCSs a full-scale ESS for wild applications such as wearable electronics, electric vehicles, large-scale facilities and so on. On account of the splendid rate property, ZHSCs can easily change service conditions as the practical current density demands change. However, the voltage window of Zn//Cap ZHSCs is still too low (lower than 1.8 V) to fulfill the demand of the practical applications. On the contrary, the Cap//ZBC ZHSCs can readily reach 2.0 V in the normal ZnSO_4_ electrolyte. By far, the reports about Cap//ZBC ZHSCs are few, thus, it is highly recommended to make some explorations on the substantial foundation of various ZBC which have been widely applied in the system of ZIBs.

**Table 1:** Electrochemical performance of reported Zn//Cap ZHSCs

Cathode	Anode	Capacity	Current density	Energy density	Peak power density	Cycle	Cycle current density	Retention (%)	Voltage (V)	Electrolyte	References
AC	2D-Zn/Ni	468 F g^−1^	0.5 A g^−1^	208 Wh kg^−1^	20,000 W kg^−1^	10,000	10 A g^−1^	99.0	0.2–1.8	1.0 M ZnSO_4_	[53]
AC	Zn foil	170 F g^−1^	0.1 A g^−1^	52.7 Wh kg^−1^	1725 W kg^−1^	20,000	2 A g^−1^	91.0	0–1.8	1.0 M Zn(CF_3_SO_3_)_2_	[101]
AC	Zn foil	121 mAh g^−1^	0.1 A g^−1^	84 Wh kg^−1^	14,900 W kg^−1^	10,000	1 A g^−1^	91.0	0.2–1.8	2.0 M ZnSO_4_	[31]
SAC^a^@CC	Zn foil	481.4 F g^−1^	0.5 A g^−1^	217 Wh kg^−1^	~ 11,000 W kg^−1^	100,000	5 A g^−1^	95.1	0–1.8	7.5 M ZnCl_2_	[170]
ZIF-8-C	Zn/ZIF-8	191 mAh g^−1^	0.5 A g^−1^	152.9 Wh kg^−1^	8000 W kg^−1^	6000	5 A g^−1^	96.0	0.2–1.8	2.0 M ZnSO_4_	[98]
HCSs	Zn/CC	86.8 mAh g^−1^	0.5 A g^−1^	59.7 Wh kg^−1^	~ 3600 W kg^−1^	15,000	1 A g^−1^	98.0	0.15–1.95	0.08 M ZnSO_4_/PAM gel	[99]
MCHS	Zn/MCHS	174.7 mAh g^−1^	0.1 A g^−1^	129.3 Wh kg^−1^	13,700 W kg^−1^	10,000	1 A g^−1^	96.0	0.2–1.8	2.0 M ZnSO_4_	[92]
MSPC^b^	Zn foil	136.5 F g^−1^	0.68 A g^−1^	36.5 Wh kg^−1^	3200 W kg^−1^	10,000	5.44 A g^−1^	85.4	0–1.8	2.0 M ZnSO_4_	[114]
NO-BPC^c^	Zn foil	51.4 mAh g^−1^	0.2 A g^−1^	48.3 Wh kg^−1^	~ 1200 W kg^−1^	90,000	3 A g^−1^	96.0	0.2–1.8	2.0 M ZnSO_4_	[115]
PCNFs^d^	Zn foil	177.7 mAh g^−1^	0.5 A g^−1^	142.2 Wh kg^−1^	15,390 W kg^−1^	10,000	10 A g^−1^	90.0	0.1–1.7	1.0 M ZnSO_4_	[111]
PCNs^e^	Zn foil	149 mAh g^−1^	0.2 A g^−1^	119 Wh kg^−1^	16,000 W kg^−1^	10,000	10 A g^−1^	91.0	0.1–1.7	1.0 M ZnSO_4_	[171]
PC800	Zn foil	179.8 mAh g^−1^	0.1 A g^−1^	104.8 Wh kg^−1^	48,800 W kg^−1^	30,000	20 A g^−1^	99.2	0–1.9	3.0 M Zn(ClO_4_)_2_	[108]
FC	Zn foil	132 mAh g^−1^	1 A g^−1^	117.5 Wh kg^−1^	16,200 W kg^−1^	10,000	5 A g^−1^	90.0	0.1–1.8	2.0 M ZnSO_4_/gelatin gel	[117]
BNO-FC^f^	Zn foil	133.5 mAh g^−1^	1 A g^−1^	119.7 Wh kg^−1^	16,500 W kg^−1^	20,000	10 A g^−1^	92.0	0.1–1.8	2.0 M ZnSO_4_/gelatin gel	[109]
NO-HPC/CC	Zn foil	138.5 mAh g^−1^	0.5 A g^−1^	110 Wh kg^−1^	20,000 W kg^−1^	10,000	5 A g^−1^	104.3	0.2–1.8	2.0 M ZnSO_4_/gelatin gel	[132]
PHCA	Zn foil	143.7 mAh g^−1^	1 A g^−1^	129.3 Wh kg^−1^	20,000 W kg^−1^	10,000	5 A g^−1^	92.0	0.1–1.8	2.0 M ZnSO_4_	[112]
PB-AC	Zn foil	169.4 mAh g^−1^	0.5 A g^−1^	169.4 Wh kg^−1^	20,000 W kg^−1^	30,000	10 A g^−1^	88.0	0.2–1.8	2.0 M ZnSO_4_	[133]
BN-CMT	Zn foil	416.6 mAh g^−1^	1 A g^−1^	472.6 Wh kg^−1^	16,000 W kg^−1^	10,000	10 A g^−1^	99.1	0.2–1.8	1.0 M ZnSO_4_ + 0.01 M ZnI_2_	[110]
BN-LDC	Zn foil	127.7 mAh g^−1^	0.5 A g^−1^	97.6 Wh kg^−1^	12,100 W kg^−1^	6500	5 A g^−1^	81.3	0.2–1.8	1.0 M ZnSO_4_/gelatin gel	[83]
N-HPC	Zn foil	136.8 mAh g^−1^	0.1 A g^−1^	191 Wh kg^−1^	3633 W kg^−1^	5000	1 A g^−1^	90.9	0.2–1.8	2.0 M ZnSO_4_	[130]
HNPC	Zn foil	177.8 mAh g^−1^	4.2 A g^−1^	107.3 Wh kg^−1^	24,900 W kg^−1^	20,000	16.7 A g^−1^	99.7	0–1.8	1.0 M ZnSO_4_	[85]
HNPC	Zn foil	148.2 mAh g^−1^	4.2 A g^−1^	91.8 Wh kg^−1^	27,600 W kg^−1^	–	–	–	0–1.8	1.0 M ZnSO_4_/PVA gel^g^	[85]
G/PANI	Zn foil	154 mAh g^−1^	0.1 A g^−1^	205 Wh kg^−1^	2455 W kg^−1^	6000	5 A g^−1^	80.5	0.3–1.6	2.0 M ZnSO_4_	[131]
MXene-rGO	Zn foil	128.6 F g^−1^	0.4 A g^−1^	34.9 Wh kg^−1^	~ 4100 W kg^−1^	75,000	5 A g^−1^	95.0	0.2–1.6	2.0 M ZnSO_4_	[139]
aMEGO	Zn foil	–	–	106.3 Wh kg^−1^	31,400 W kg^−1^	80,000	8 A g^−1^	93.0	0–1.9	3.0 M Zn(CF_3_SO_3_)_2_	[82]
rGO/CNT	Zn/C	104.5 F cm^−3^	0.4 A cm^−3^	48.5 mWh cm^−3^	3599 mW cm^−3^	10,000	3.2 mA cm^−3^	98.5	0–1.8	2.0 M ZnSO_4_/PAA gel^h^	[120]
rGO/CNT	Zn/C	84.7 F cm^−3^	0.4 A cm^−3^	–	–	10,000	3.2 mA cm^−3^	86.2	0–1.8	2.0 M ZnSO_4_/PVA gel	[120]
Ti_3_C_2_	Zn/Ti_3_C_2_	132 F g^−1^	0.5 A g^−1^	–	–	1000	3 A g^−1^	82.5	0.1–1.35	1.0 M ZnSO_4_/gelatin gel	[96]
3DP-Ti_3_C_2_	Zn/CNT	244.6 mF cm^−2^	10 mA cm^−2^	0.10 mWh cm^−2^	5.9 mW cm^−2^	6000	10 mA cm^−2^	86.5	0.1–1.2	2.0 M ZnSO_4_	[91]
3DP-Ti_3_C_2_	Zn foil	~ 1 F cm^−2^	0.1 A g^−1^	0.17 mWh cm^−2^	21 mW cm^−2^	4000	10 A g^−1^	78.0	0.1–1.2	2.0 M ZnSO_4_	[91]
Ti_3_C_2_T_*x*_	Zn/Ti_3_C_2_T_*x*_	72 mF cm^−2^	10 mV s^−1^	0.02 mWh cm^−2^	5.0 mW cm^−2^	50,000	–	79.6	0–1.4	6.0 M ZnCl_2_/PVA gel	[153]
rGO/PPD	Zn foil	~ 3 F cm^−2^	1 mA cm^−2^	1.1 mWh cm^−2^	8.0 mW cm^−2^	4000	7 mA cm^−2^	100.0	0.2–1.8	1.0 M Zn(AC)_2_	[140]
PAC^i^	Zn foil	315 mAh g^−1^	0.19 A g^−1^	195 Wh kg^−1^	10,000 W kg^−1^	10,000	10 A g^−1^	91.0	0.1–1.8	2.0 M ZnSO_4_/PVA gel	[77]
PDA/PCC	Zn foil	1.25 mAh cm^−2^	1 mA cm^−2^	1.06 mWh cm^−2^	8.5 mW cm^−2^	10,000	10 mA cm^−2^	100.0	0.1–1.8	2.0 M ZnSO_4_	[154]
TDP/AC^j^	Zn foil	1.16 mAh cm^−2^	1 mA cm^−2^	1.03 mWh cm^−2^	9.0 mW cm^−2^	2000	8 mA cm^−2^	71.0	0.1–1.9	2.0 M ZnSO_4_/gelatin gel	[172]
TiN	Zn plate	489.8 F g^−1^	0.2 A g^−1^	135 Wh kg^−1^	3472 W kg^−1^	10,000	1 A g^−1^	~ 78.0	0.1–1.9	1.0 M ZnSO_4_	[169]
RuO_2_·H_2_O	Zn foil	122 mAh g^−1^	0.1 A g^−1^	119 Wh kg^−1^	16,740 W kg^−1^	10,000	20 A g^−1^	87.5	0.4–1.6	2.0 M Zn(CF_3_SO_3_)_2_	[165]

^a^*SAC* silk-derived activated carbon

^b^*MSPC* molten salt porous carbon

^c^*NO-BPC* N, O co-doped bamboo porous carbon

^d^*PCNF* porous carbon nanoflakes

^e^*PCNs* porous carbon nanosheets

^f^*BNO-FC* B, N, O co-doped flower-like carbon

^g^*PVA* polyvinyl alcohol

^h^*PAA* polyacrylic acid

^i^*PAC* poly(1,5-NAPD)/AC

^j^*TDP/AC* poly(4,4’-thiodiphenol, TDP)/AC

### Cap//ZBC ZHSCs

Generally, the ZHSCs are constructed by the integration of ZIBs and supercapacitors, where the Cap electrodes are typically carbon and pseudocapacitive electrodes as we reviewed in Sect. 2.2, the battery-type electrodes are ZBC and Zn anodes in the ZIB systems. Before this section, we amply discussed the Zn//Cap ZHSCs, which comprise Zn anodes and Cap cathodes. Conversely, the design theory of Cap//ZBC ZHSCs is to replace the Zn anode in ZIBs with Cap electrodes. Hence, different from the Zn//Cap ZHSCs, the Cap electrodes will act as the anode and Zn-ion battery-type materials will act as the cathode in the Cap//ZBC ZHSCs. Notably, many potential ZBC of ZHSCs have been reported in the ZIBs, such as manganese-based oxide, vanadium-based oxides, prussian blue and analogues, spinels, etc.

**Fig. 7:**
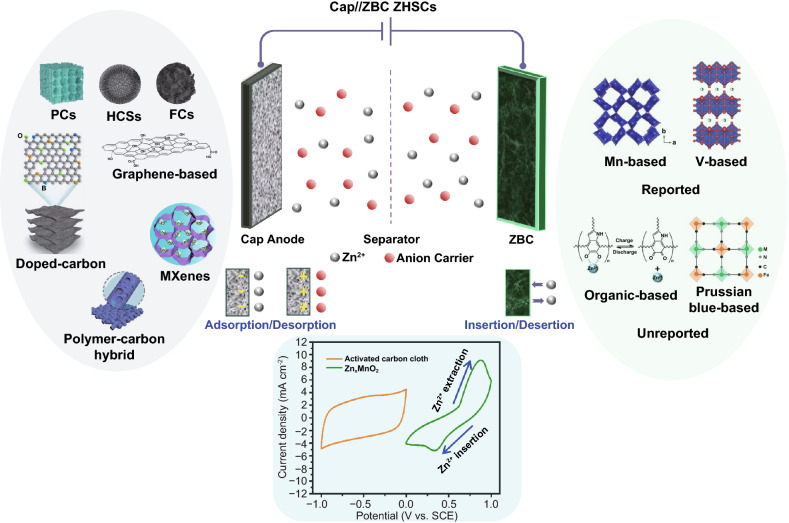
Schematic illustration (Cap anodes, ZBC, energy storage mechanisms and CV behaviors) of Cap//ZBC ZHSCs

As illustrated in Fig. 7, the Cap anodes, ZBC, energy storage mechanism and CV behavior of Cap//ZBC ZHSCs are exhibited. Same as the Zn//Cap ZHSCs, the electrolytes are Zn salt solutions. Basically, Cap electrodes perform the same energy storage mechanism in the Zn//Cap ZHSCs, but act as anode opposed to the ZBC. The CV curve of ACC anodes in Fig. 7 presented a typical rectangular shape of Cap anodes. On the contrary, the CV plot of Zn_*x*_MnO_2_ cathodes showed two obvious redox peaks at approximately 0.33 V/0.88 V (*vs*. SCE), corresponding to the insertion/desertion reaction processes of ZBC. The Zn-ion insertions happen during the discharging processes of ZHSCs, while the Zn-ion desertions occur during the charging periods. Gratifyingly, many ZBC can reversibly proceed insertion/extraction reactions of Zn-ions, which have been widely confirmed in ZIBs, including manganese oxides (MnO_2_ with a different crystalline structure, Mn_2_O_3_, etc.) [87, 173], vanadium oxides (V_2_O_5_, V_2_O_7_, V_3_O_8_, and homologous derivatives, etc.) [174–177], prussian blue and analogues [8, 178–180] and spinels (ZnMn_2_O_4_, etc.) [181–184]. Although the ionic radius of Zn-ion is small (0.75 Å), the hydrated ionic radius of Zn-ion (3.40—3.82 Å) raises strong demand for suitable insertion cathode materials of ZHSCs [87]. Some special crystal structures like layered structure (*δ*-MnO_2_, V-based materials, etc.), tunnel structure (*α*-MnO_2_, *γ*-MnO_2_, etc.) and framework (prussian blue and analogues, spinels, etc.) can effectively store Zn-ion via reversible insertion/desertion processes. A large and stable crystal structure can store a great number of Zn-ion and then provide considerable capacity by reversible chemical redox reactions between Zn-ion and cathode materials.

When the ZHSCs are assembled based on Cap anodes and ZBC, the Cap//ZBC ZHSCs can provide a higher voltage window of 0–2.0 V and more possibilities like Zn-dendrite-free ZHSCs. However, only a few ZBC (e.g., manganese-based oxides and vanadium-based oxides) have been applied in this configuration of ZHSCs and many problems still exist. Here, we will summarize some reported studies of Cap//ZBC ZHSCs in the following review, trying to draw more attention to the ZBC and the second electrode configuration of Cap//ZBC ZHSCs.

#### Manganese-Based Oxides

Manganese oxides, with many outstanding properties like low cost, high theoretical capacity, tunable and multifarious crystal structure, etc., have been well investigated in various ESSs [185–187]. Among various manganese oxides, MnO_2_ is one of the most promising manganese oxides. By linking the fundamental units MnO_6_ octahedra with shared edges and vertices, tunnel-type *α*-MnO_2_, *β*-MnO_2_, *δ*-MnO_2_, *ε*-MnO_2_ and layered *γ*-MnO_2_ are obtained [87]. Generally, many MnO_2_ cathodes with diverse crystal structures have been employed in ZIBs while only a few were reported in ZHSCs.

**Fig. 8:**
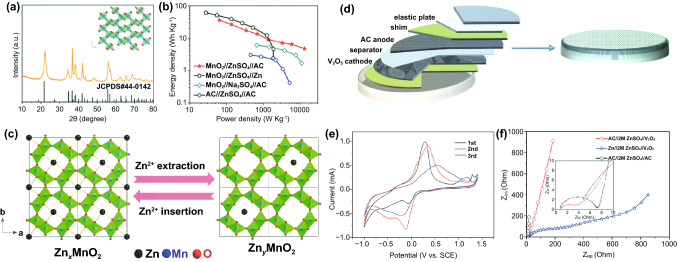
**a** XRD pattern and crystal structure (inset) of *γ*-MnO_2_ cathodes. **b** Ragone plots of various *γ*-MnO_2_-based ESSs. Reproduced with permission from Ref. [188]. Copyright 2019, Elsevier. **c** Schematic illustration of the Zn-ion storage mechanisms in the tunnel-structure Zn_*x*_MnO_2_ cathode. Reproduced with permission from Ref. [34]. Copyright 2020, Wiley–VCH. **d** Full cell schematic of AC//2.0 M ZnSO_4_//V_2_O_5_ ZHSCs. **e** CV curves tested by three-electrode system of V_2_O_5_. **f** Nyquist plots of relevant devices, including ZHSC, ZIB and supercapacitor. Reproduced with permission from Ref. [189]. Copyright 2019, Springer Nature

As the pioneer of the Cap//ZBC ZHSCs, Kang group reported the AC//*γ*-MnO_2_ ZHSCs with a wide voltage window of 0–2.0 V, the as-assembled ZHSCs showed a capacity of 54.1 mAh g^−1^ in 2.0 M ZnSO_4_ electrolyte while exhibited a capacity of 83.8 mAh g^−1^ in the 2.0 M ZnSO_4_ and 0.5 M MnSO_4_ electrolyte [188]. The XRD pattern and tunnel crystal structure of *γ*-MnO_2_ cathode are displayed in Fig. 8a, the insertion/desertion processes of Zn-ion occur in the tunnel structure of *γ*-MnO_2_ cathodes. However, due to the solubility and poor electrical conductivity of Mn-based materials, the AC//*γ*-MnO_2_ ZHSCs can merely keep 65.3% of initial capacity after 3000 cycles without the MnSO_4_ additive electrolyte. When changing the electrolyte from ZnSO_4_ to Zn(CF_3_SO_3_)_2_, the cycling performance of AC//*γ*-MnO_2_ ZHSCs can be tremendously improved to 93.4% after longer 5000 cycles. Generally, the CF_3_SO_3_^−^ anion results in lower charge transfer resistance and higher ion diffusion rate than SO_4_^2−^ anion. Furthermore, the by-products are not visibly detected in the Zn(CF_3_SO_3_)_2_ electrolyte due to the reduced interaction between Zn-ions and adjacent water molecules [82]. When compared with the Zn//*γ*-MnO_2_ ZIBs (Fig. 8b), the AC//*γ*-MnO_2_ ZHSCs presented a higher power density of 13,000 W kg^−1^ and the poor rate performance of *γ*-MnO_2_-based ZIBs is greatly enhanced in the fast ZHSCs. Although the capacity of AC//*γ*-MnO_2_ ZHSCs is still unsatisfactory, the strengths of Cap//ZBC ZHSCs like larger voltage window, high power density, excellent rate performance and unique energy storage mechanisms are presented.

In addition, Mai group recently investigated the application of tunnel-type *α*-MnO_2_ (Zn_*x*_MnO_2_) and layered *δ*-MnO_2_ cathodes in ZHSCs, the active carbon cloth (ACC)//Zn_*x*_MnO_2_ and ACC//*δ*-MnO_2_ ZHSCs are prepared and tested [34]. As depicted in Fig. 8c, some pre-intercalation Zn-ions are stored in the tunnel structure of Zn_*x*_MnO_2_ cathode before electrochemical tests. Additionally, the Zn-ions are extracted from the tunnel structure when the ZHSCs are charging and conversely, inserted into the tunnel structure during discharging periods. The capacity of MnO_2_ cathodes may fluctuate a lot at first few cycles since a great number of Zn-ions are left in the tunnel structure, which leads to the unbalanced Zn-ion insertion/desertion reactions. However, depend on the pre-intercalation Zn-ion in the tunnel structure, the Zn_*x*_MnO_2_ cathode is stabilized in advance and then performed steady cycling performance. Compared with the tunnel structure of *γ*-MnO_2_ (1 × 1 and 1 × 2 tunnel structure: 2.3 × 2.3 Å and 2.3 × 4.6 Å), the *α*-MnO_2_ (2 × 2 tunnel structure: 4.6 × 4.6 Å) possess a larger and more suitable tunnel structure for Zn-ions storage due to the close hydrated ionic radii of Zn-ions (4.3 Å). Accordingly, the ACC//Zn_*x*_MnO_2_ ZHSCs remained 83.1% of initial capacity after 5000 cycles, and the ex-situ XRD spectra of Zn_*x*_MnO_2_ cathode presented no evident excursion during the charging/discharging processes. Layered *δ*-MnO_2_ is also a promising Zn-ion storage cathode with an ultra-large interlayer spacing of ~ 7 Å. Nevertheless, the structure of *δ*-MnO_2_ is prone to collapse in sulfate solution during the insertion/desertion processes of Zn-ions, thus, the capacity retention of ACC//*δ*-MnO_2_ ZHSCs quickly fade to 83.2% after 10 cycles and then drop to 70.4% after 35 cycles, finally end at 45.3% after 5000 cycles. Hence, it is believed that the Zn-ion pre-intercalation can be a way to promote the stability of manganese-based cathodes. The ACC//Zn_*x*_MnO_2_ ZHSCs delivered an ultra-high and stable areal capacity of 1745.8 mF cm^−2^ at 2 mA cm^−2^. Meanwhile, ACC//Zn_*x*_MnO_2_ ZHSCs performed a wide operating voltage window of 0–2.0 V and excellent areal power density of 20.1 mW cm^−2^, which is vital for many flexible and wearable electronic devices.

Notably, a novel ZHSC of Ti_3_C_2_T_*x*_//MnO_2_-CNTs was proposed by Wang et al*.*, the pseudocapacitive MXene materials act as the Cap anode and the battery-type MnO_2_-CNTs act as the cathode [190]. The electrode properties were demonstrated by the three-electrode system, where the MnO_2_-CNTs presented typical charge/discharge platforms in the GCD curves and a couple of redox peaks were detected in the CV curves. On the contrary, the Ti_3_C_2_T_*x*_ anode showed distinct Cap behaviors with straight up and down GCD curves. These results confirmed that the Zn-ion storage mechanism of ZBC is pretty different from the pseudocapacitive electrode. The pseudocapacitive TMCs should not be confused with TMCs ZBC. Benefit from the balanced electrode configuration of Cap//ZBC ZHSCs and excellent electrochemical performance of MnO_2_, the Ti_3_C_2_T_*x*_//MnO_2_-CNTs ZHSCs exhibited splendid capacity of 115.1 F g^−1^ as well as remarkable longevity of 83.6% capacity retention after 15,000 cycles, which is the best cycling performance in the system of Cap//ZBC ZHSCs.

For many manganese-based oxide cathodes, the crystal transformations will occur during the insertion/desertion processes of different cations or water molecules, and different structures of manganese-based oxide cathodes may lead to different Zn-ion storage performance in ZHSCs. By far, the *α*-MnO_2_ exhibited a prominent electrochemical performance as well as stable tunnel structure among various manganese-based oxide cathodes. Although numerous advantages the manganese-based oxide cathodes have shown, the poor rate capability, easy-dissolution in electrolyte and relatively unstable structure are still challenges in the application of Cap//manganese oxide ZHSCs. Hence, more efforts should be devoted to investigating suitable manganese oxide cathodes for high-performance ZHSCs and create more possibility for Cap//ZBC ZHSCs.

#### Vanadium-Based Oxides

Apart from the manganese-based oxides, vanadium-based oxides are another widely applied ZBC for ZHSCs. With high theoretical capacity, low cost and stable layer crystal structure, vanadium-based oxide cathodes are suitable for multivalent ions storage [191, 192]. However, by far, only one research has been reported about the ZHSCs based on the vanadium-based oxide cathodes [189]. Therefore, in this section, the rare vanadium-based ZHSC will be discussed in detail.

As illustrated in Fig. 8d, Ma et al*.* employed V_2_O_5_ materials as the cathode of ZHSCs and assembled the AC//V_2_O_5_ ZHSCs where the V_2_O_5_ materials and AC powder act as the cathode and anode, respectively [189]. Typically, V_2_O_5_ cathodes tend to proceed an activated process before longtime charging/discharging according to the CV curves shown in Fig. 8e, where the redox peaks of V_2_O_5_ cathode sharply changed at the first three cycles. It is believed that the incipient charging/discharging process of V_2_O_5_ cathode leads to structural transformations, and the V_2_O_5_ cathodes turn out to be stable after activation. The possible electrochemical reaction is as the following Eq. (2-2):

2-2 $${\text{V}}_{{2}} {\text{O}}_{{5}} + x{\text{Zn}}^{{{2} + }} \leftrightarrow {\text{ Zn}}_{x} {\text{V}}_{{2}} {\text{O}}_{{5}}\, \left( {x \approx \, 0.{3}} \right)$$

Similarly, the AC//V_2_O_5_ ZHSCs can also achieve a wide voltage window of 0–2.0 V, which is unreachable for vanadium-based ZIBs (most < 1.4 V). Furthermore, based on the EIS results of various ESSs (Fig. 8f), the AC//V_2_O_5_ ZHSCs showed the smaller charge transfer resistance and higher ion diffusion rate than Zn//V_2_O_5_ ZIBs and normal AC//AC supercapacitors. Thus, ZHSCs present superiority in comparison with ZIBs and supercapacitors. However, the AC//V_2_O_5_ ZHSCs only provided a limited capacity of 57.4 mAh g^−1^ and an energy density of 34.6 Wh kg^−1^. Also, the ZHSCs cannot maintain appropriate coulombic efficiency at an ultra-low current density of 0.1 A g^−1^, which can be attributed to the polarization of V_2_O_5_ at 1.8–2.0 V. Gratifyingly, the cycling stability test of AC//V_2_O_5_ ZHSCs is quite surprised, which presented a capacity retention of 97.3% after 6000 cycles at relatively low cycling current density of 0.5 A g^−1^, indicating that the V_2_O_5_ cathodes possess a suitable and stable structure for ZHSCs. As we all know, the excellent performance of Cap//ZBC ZHSCs is generally determined by the ZBC used. However, only one work about vanadium-based cathode of ZHSCs has been reported, moreover, the Zn-ions storage mechanism of vanadium-based ZHSCs is still ambiguous and the capacity of vanadium-based ZHSCs is unsatisfied. Therefore, more efforts should be devoted to promoting the development of vanadium-based oxide cathodes for high-performance ZHSCs.

As summarized in Table 2, the Cap//ZBC ZHSCs presented prominent characteristics like high voltage window, abundant electrode collocations and special properties depend on various ZBC. More significantly, the Zn-foil-free ZHSCs can avoid the potential safety hazard of Zn dendrite, leading to a Zn-dendrite-free ZHSC. Although the electrochemical performance of Cap//ZBC ZHSCs is not enough for practical applications, we believe that after sufficient investigations and creations, the Cap//ZBC ZHSCs will perform splendid electrochemical performance which may even superior to the Zn//Cap ZHSCs. To sum up, the Cap//ZBC ZHSCs still have many difficulties that need to conquer, here we summarize as the following aspects: (1) relatively low energy density, (2) inferior rate performance and (3) weak cycling stability, particularly the MnO_2_ cathodes which are easy to dissolve. Nevertheless, the in-depth understandings of energy storage mechanisms of various ZBC are plenarily needed, where the mechanisms and extent of reactions may differ from the conditions in ZIBs. The design theory of ZBC for high-performance ZHSCs should be high porosity, HSSA, stable crystal structure (Zn^2+^-adapted) and excellent electrical conductivity. By designing ZBC in a way of the Cap electrode may greatly enhance the rate performance and cycling performance of the Cap//ZBC ZHSCs. Momentously, it is strongly believed that with more attempts and investigations, the electrochemical performance of Cap//ZBC ZHSCs will be enhanced and then become a promising candidate for next-generation ESSs.

**Table 2:** Electrochemical performance of reported Cap//ZBC ZHSCs

Cathode	Anode	Capacity	Current density	Energy density	Peak power density	Cycle	Cycle current density	Retention (%)	Voltage (V)	Electrolyte	References
Zn_*x*_MnO_2_	ACC	1746 mF cm^−2^	2 mA cm^−2^	969.9 μWh cm^−2^	20.1 mW cm^−2^	5000	15 mA cm^−2^	83.1	0–2.0	2.0 M ZnSO_4_ + 0.4 M MnSO_4_	[34]
*δ*-MnO_2_	ACC	1420 mF cm^−2^	2 mA cm^−2^	789.1 μWh cm^−2^	20.0 mW cm^−2^	5000	15 mA cm^−2^	45.3	0–2.0	2.0 M ZnSO_4_ + 0.4 M MnSO_4_	[34]
Zn_*x*_MnO_2_	ACC	1.446 F cm^−2^	1 mA cm^−2^	803.6 μWh cm^−2^	10.0 mW cm^−2^	–	–	–	0–2.0	2.0 M ZnCl_2_ + 0.4 M MnSO_4_/PVA gel	[34]
MnO_2_-CNTs	Ti_3_C_2_T_*x*_	115.1 F g^−1^	1 mV s^−1^	98.6 Wh kg^−1^	2480.6 W kg^−1^	15,000	5.2 A g^−1^	~ 83.6	0–1.9	2.0 M ZnSO_4_ + 0.1 M MnSO_4_	[190]
MnO_2_	AC	54.1 mAh g^−1^	0.1 A g^−1^	34.8 Wh kg^−1^	13,000 W kg^−1^	3000	–	65.3	0–2.0	2.0 M ZnSO_4_	[188]
MnO_2_	AC	83.8 mAh g^−1^	0.1 A g^−1^	58.6 Wh kg^−1^	–	5000	~ 0.2 A g^−1^	61.8	0–2.0	2.0 M ZnSO_4_ + 0.5 M MnSO_4_	[188]
MnO_2_	AC	46.4 mAh g^−1^	0.1 A g^−1^	29.5 Wh kg^−1^	–	5000	~ 0.2 A g^−1^	93.4	0–2.0	2.0 M Zn(CF_3_SO_3_)_2_	[188]
V_2_O_5_	AC	57.4 mAh g^−1^	0.1 A g^−1^	34.6 Wh kg^−1^	1300 W kg^−1^	6000	0.5 A g^−1^	97.3	0–2.0	2.0 M ZnSO_4_	[189]

## Energy Storage Mechanisms of Cap Electrodes

Importantly, Cap electrodes perform a vital role in both two configurations of ZHSCs, however, the energy storage mechanisms of Cap electrodes are pretty intricate according to the reported ZHSCs [31, 188, 193]. Besides the simple EDLC of the physical adsorption/desorption processes of cations and anions, more results are proved to confirm extra energy storage mechanisms of Cap electrodes, including precipitation/dissolution and chemical pseudocapacitive reactions. Hence, the related investigations of Cap electrodes will be concluded in the following section [31, 85, 188]. As the fundamental energy storage mechanism, the physical adsorption/desorption processes are presented as following Eqs. (3-1, 3-2), where the X^−^ represents the monovalent anion (Cl^−^, NO_3_^−^, CF_3_SO_3_^−^ and CH_3_COO^−^), the Y^2^^−^ represents the divalent anion (SO_4_^2−^) [132]:

3-1 $${\text{C }} + {\text{ Zn}}^{{{2} + }} /{\text{H}}^{ + } \leftrightarrow {\text{ C }}||{\text{ Zn}}^{{{2} + }} /{\text{H}}^{ + }$$

3-2 $${\text{C }} + {\text{ X}}^{ - } /{\text{Y}}^{{{2} - }} \leftrightarrow {\text{ C }}||{\text{ X}}^{ - } /{\text{Y}}^{{{2} - }}$$

### Precipitation/Dissolution of By-Products

Typically, the precipitation/dissolution processes of by-products like Zn_4_SO_4_(OH)_6_·nH_2_O have been found in the system of ZIBs, especially in the Mn-based and V-based ZIBs [87]. The by-products with loose and porous layers will randomly form on the surface of bare Zn foil during charging/discharging processes, which leads to the uneven surface and Zn dendrite, then the poor cycling performance of ZIBs [194, 195]. Therefore, the investigation of formation mechanisms of the by-products in ZHSCs is more necessary since the Zn anode of ZHSCs can be greatly consumed by the fast charging/discharging processes. Furthermore, the formation of by-products also happened on the Cap electrodes of ZHSCs, thus, the dual formation of by-products may affect the capacity and cycling performance of ZHSCs during charging/discharging processes.

**Fig. 9:**
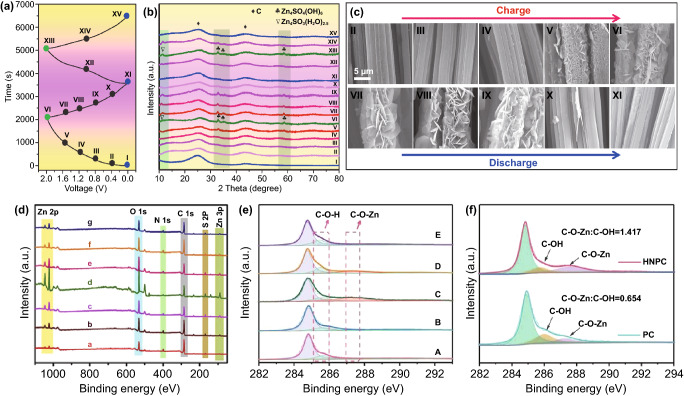
**a** GCD profiles of ACC//Zn_*x*_MnO_2_ ZHSCs at 2 mA cm^−2^. **b** Corresponding ex-situ XRD patterns and **c** SEM images of ACC anodes at different charge/discharge states. Reproduced with permission from Ref. [34]. Copyright 2020, Wiley–VCH. **d** Ex-situ XPS spectra of carbon cathodes at various states, discharging from **a** to **d** and charging from **d** to **g**. Reproduced with permission from Ref. [77]. Copyright 2020, Elsevier. **e** Ex-situ C 1 s XPS spectra of HNPC cathodes, discharging from A to C and charging from C to E. **f** C 1 s XPS spectra of PC and HNPC cathodes at 0 V. Reproduced with permission from Ref. [85]. Copyright 2019, Wiley–VCH

In the ZHSCs systems, Kang group demonstrated the existence of by-products of Zn_4_SO_4_(OH)_6_·5H_2_O on both AC cathode and Zn anode in ZnSO_4_ electrolyte after discharging processes (from 1.8 to 0.2 V) [31]. Notably, part of by-products is left on the surface of AC cathode after 10 cycles, indicating the irreversible reactions of precipitation/dissolution. Furthermore, the formation of by-products is also detected in other ZHSCs and even in the Cap//ZBC ZHSCs. Particularly, Mai group reported that the by-products of Zn_4_SO_4_(OH)_6_·nH_2_O and ZnSO_3_·2.5(H_2_O) were also observed on the surface of ACC in ACC//Zn_*x*_MnO_2_ ZHSCs [34]. The ex-situ XRD spectra based on ACC//Zn_*x*_MnO_2_ ZHSCs (Fig. 9a, b) clearly displayed XRD peaks of Zn_4_SO_4_(OH)_6_·nH_2_O and ZnSO_3_·2.5(H_2_O) at state VI and XIII, which correspond to the voltage of 2.0 V. The by-products gradually precipitate during the charging process and dissolve during discharging period. Typically, this process was further demonstrated by the ex-situ SEM images shown in Fig. 9c. Also, the by-product of Zn_4_SO_4_(OH)_6_·0.5H_2_O is detected by Kang group on the surface of ZBC (*γ*-MnO_2_) [196]. On the contrary, the precipitation/dissolution processes are inverse since the Cap electrodes are applied as the cathode in the Zn//Cap ZHSCs, which was proved by the ex-situ XPS spectra of carbon cathode depicted in Fig. 9d [77]. The Zn, O, S signals, which correspond to the formation of by-product Zn_4_SO_4_(OH)_6_·nH_2_O, are enhanced by degrees during discharging process while the signals decreased during the charging process. Additionally, apart from the Cap electrodes, Zn_4_SO_4_(OH)_6_·5H_2_O can also be detected on the surface of Zn anodes [31, 82]. Typically, the reaction equation of by-products can be described as following Eqs. (3-3, 3-4) [34, 188]:

3-3 $${\text{4Zn}}^{{{2} + }} + {\text{ 6OH}}^{ - } + {\text{ SO}}_{{4}}^{{{2} - }} + {\text{ nH}}_{{2}} {\text{O }} \leftrightarrow {\text{ Zn}}_{{4}} \left( {{\text{OH}}} \right)_{{6}} {\text{SO}}_{{4}} \cdot {\text{nH}}_{{2}} {\text{O}}$$

3-4 $${\text{5Zn}}^{{{2} + }} + {\text{ 4OH}}^{ - } + {\text{ 2SO}}_{{4}}^{{{2} - }} + {\text{ 4H}}_{{2}} {\text{O }} + {\text{ 2e}}^{ - } \leftrightarrow {\text{ Zn}}_{{4}} \left( {{\text{OH}}} \right)_{{6}} {\text{SO}}_{{4}} \cdot 0.{\text{5H}}_{{2}} {\text{O }} + {\text{ ZnSO}}_{{3}} \cdot {2}.{5}\left( {{\text{H}}_{{2}} {\text{O}}} \right)$$

Significantly, recently reported works ascribe this phenomenon to the change of pH value. As we know about the faintly acid property (initial pH of ~ 4.5) of ZnSO_4_ electrolyte, by-product formed when the pH value becomes higher than ~ 5.3 and then dissolved when pH decreases [31]. Dong et al*.* supposed that the pH change results from the reversible hydrogen storage in nano-porous AC at a low potential, presented as Eqs. (3-5, 3-6) [35, 108]. Furthermore, Kang group particularly summarized two possible ways of causing the increase of pH in Cap//ZBC ZHSCs: (1) H^+^-decrease: the reaction between H^+^ and MnO_2_ whereas the reaction is not dominant, (2) OH^−^-increase: water decomposition in nano-porous AC surface like Eq. (3-6) [188]. Typically, Zhang group found that the competitive hydrogen evolution process coexisted with Zn plating in ZnSO_4_ electrolyte below 0.2 V which can be the reason for pH increase [82]. Significantly, in our previous work of the ACC//Zn_*x*_MnO_2_ ZHSCs, ACC acts as anode and the deposition of Zn_4_SO_4_(OH)_6_·nH_2_O or ZnSO_3_·2.5(H_2_O) appeared only at the high voltage stage and then dissolved at low voltage (shown in Fig. 9c), it is believed that the high voltage can attract Zn^2+^ in ZnSO_4_ electrolyte and further form by-products of Zn_4_SO_4_(OH)_6_·nH_2_O and ZnSO_3_·2.5(H_2_O) [34]. Briefly, the alteration of pH value is the outward manifestation and can be instructional for theoretical researches, more importantly, the hydroxyl/hydrogen ions and the adsorption of electrodes to the inverse cation/anion are the inner cause and mechanism of the precipitation/dissolution processes. Herein, we conclude this phenomenon as following results: (1) reactions in both cathodes and anodes which lead to the pH change, (2) the attraction to Zn^2+^/SO_4_^2−^ at different voltage stages in two configurations of ZHSCs. Typically, Eqs. (3-5, 3-6) are shown as follow [35, 108]:

3-5 $${\text{C }}{-}{\text{ OH}} \leftrightarrow {\text{C }} = {\text{ O }} + {\text{ H}}^{ + } + {\text{ e}}^{ - }$$

3-6 $${\text{AC }} + {\text{ H}}_{{2}} {\text{O }} + {\text{ e}}^{ - } \leftrightarrow {\text{AC}} \cdot {\text{H }} + {\text{ OH}}^{ - }$$

The influence of the by-products’ formation can be divided into two steps. At first, the surface of electrodes is clean and easy to access electrolyte for energy storage processes. In this case, the pH value of the electrolyte stays low and the slow formation process of by-products can provide considerable capacity for the ZHSCs. However, after a short cycle, the reactions between the H^+^ of electrolyte and electrodes increase the pH value of electrolyte, and then lead to the fast and uncontrollable formation process of by-products. The uneven precipitation/dissolution process can greatly change the morphology of electrodes. Hence, due to the irreversible formation process of by-products, the ZHSCs can offer an excellent capacity at the first few cycles but suffer from the morphology modification for long cycles.

### Chemical Pseudocapacitive Reactions

Only with the physical adsorption/desorption and precipitation/dissolution of by-products, there is no reason that the Cap electrodes are able to provide such high capacity which can even reach the capacity of whole redox-reaction-based batteries. When adopting the strategies of dopant, polymer-carbon hybrid and other activated processes, the Cap electrodes are equipped with pseudocapacitive properties due to the functional group in the carbon skeleton and active structure/materials. The small redox peaks of Cap electrodes can be observed in the CV curves of many reported ZHSCs [77, 92, 108].

Significantly, Lu group captured more chemical transformation details of Cap electrodes which are depicted in Fig. 9e [85]. When the carbon electrodes were investigated by ex-situ XPS, the intensity of the C–OH peak keeps decreasing from 1.8 to 0 V (A to C, discharging process), and increasing from 0 to 1.8 V (C to E, charging process) whereas the C–O–Zn peak is reversed, indicating that there are reversible pseudocapacitive chemical reactions between the C–OH and C–O–Zn bonds. Namely, the C–OH bonds are chemically associated with Zn-ion to form C–O–Zn bonds, which is the determining step in the entire chemical reactions. Typically, the ratio of the C–O–Zn to C–OH at 0 V is generally related to the electrochemical performance, implying the extent of pseudocapacitive reactions between C–OH and Zn-ion. As shown in Fig. 9f, the ratio of HNPC electrode is ~ 2.2 times higher than the ratio of PC electrode which is close to the ratio of capacity (~ 2.6 times), indicating the highly correlative relationship between the chemical pseudocapacitive reactions and ZHSCs capacity. Besides the Zn-ions in the electrolyte, H^+^ ions are also demonstrated to be a part of these reactions [77, 132]. Due to the faintly acid property of ZnSO_4_ electrolyte, H^+^ ions generally exist in the electrolyte and take part in the chemical pseudocapacitive reactions of Cap electrodes and contribute considerable capacity to the ZHSCs. Meanwhile, it is also a crucial reason that the OH^−^ concentration of electrolyte will increase, and further results in the precipitation/dissolution processes of by-products on the surface of various electrodes. The chemical pseudocapacitive reactions are presented as the following Eq. (3-7), and when N, B or other heteroatoms substitute part of C atoms in the carbon skeleton, the reactions proceed as Eq. (3-8), where the X represents element N, B or other heteroatoms:

3-7 $${\text{C }}{-}{\text{ OH }} + {\text{ Zn}}^{{{2} + }} /{\text{H}}^{ + } + {\text{ e}}^{ - } \leftrightarrow {\text{ C}} \cdots {\text{O}} \cdots {\text{Zn}}/{\text{C}} \cdots {\text{O}} \cdots {\text{H}}$$

3-8 $${\text{X }}{-}{\text{ OH }} + {\text{ Zn}}^{{{2} + }} /{\text{H}}^{ + } + {\text{ e}}^{ - } \leftrightarrow {\text{ X}} \cdots {\text{O}} \cdots {\text{Zn}}/{\text{X}} \cdots {\text{O}} \cdots {\text{H}}$$

In short, these reactions enable considerable capacity and abundant properties of Cap electrodes to realize better ZHSCs. Meanwhile, the combination of various energy storage mechanisms in Cap electrodes has been carefully analyzed. Even though, we deem it is necessary to further investigate and review the energy storage mechanisms of Cap electrodes. The kernel of the researches should be the balance between energy density and longevity, where the energy storage mechanisms can be the internal reasons for the external electrochemical performance.

## Electrolyte

### Effects of Various Electrolyte Anions

As the most frequently used anode in the Zn//Cap ZHSCs, Zn foils anodes possess similar size and thickness, but the corresponding Zn-ion storage behaviors are diverse in different electrolytes [82]. The depositing/stripping behaviors of Zn anodes are highly related to the anions applied in the electrolytes. Generally, the cation is Zn^2+^ while the anions are diversiform, including Cl^−^ [34, 153], SO_4_^2−^ [31, 53], CF_3_SO_3_^−^ [101], ClO_4_^−^ [108], NO_3_^−^ and CH_3_COO^−^ [140, 170], the interaction between the water molecules and electrolyte anions will affect the Zn depositing/stripping reactions and further determine the Zn-ion storage efficiency as well as the electrochemical performance of ZHSCs. Here, we briefly review some reported researches about the effects of various electrolytes on ZHSCs [82].

**Fig. 10:**
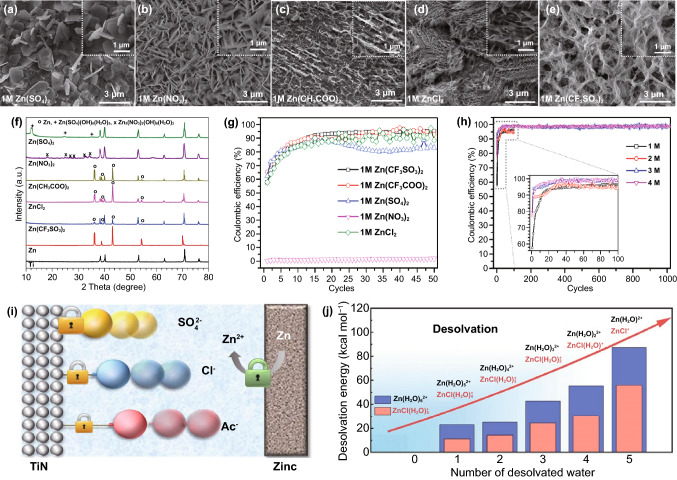
**a–e** SEM images of pristine Zn and Ti foils with Zn layer plated in various electrolytes. **f** Corresponding XRD patterns and **g** coulombic efficiencies (CEs) of Zn depositing/stripping in various electrolytes. **h** CEs in Zn(CF_3_SO_3_)_2_ electrolytes with different concentrations. Reproduced with permission from Ref. [82]. Copyright 2019, Wiley–VCH. **i** Schematic illustration of self-discharge behaviors of Zn//TiN ZHSCs in three different electrolytes. Reproduced with permission from Ref. [169]. Copyright 2021, Wiley–VCH. **j** Desolvation energies of [Zn(H_2_O)_6_]^2+^ and [ZnCl(H_2_O)_5_]^+^. Reproduced with permission from Ref. [170]. Copyright 2021, Wiley–VCH

As the exhibited SEM images and XRD spectra in Fig. 10a–f, Wu et al*.* investigated the plating morphology and phase composition of Zn–Ti (Ti served as the substrate) anodes in five different electrolytes of 1.0 M ZnSO_4_, Zn(NO_3_)_2_, Zn(CH_3_COO)_2_, ZnCl_2_ and Zn(CF_3_SO_3_)_2_ [82]. Surprisingly, the by-products of Zn_4_(SO_4_)(OH)_6_⋅5H_2_O and Zn_5_(NO_3_)_2_(OH)_8_⋅2H_2_O were detected on the Ti substrates in ZnSO_4_ and Zn(NO_3_)_2_ electrolytes, respectively. No redundant peaks are observed in the XRD spectra of Zn anode when employed the Zn(CH_3_COO)_2_, ZnCl_2_ and Zn(CF_3_SO_3_)_2_ as the electrolyte. Correspondingly, the SEM images of Zn–Ti anodes in different electrolytes can be divided into two types. In the ZnSO_4_ and Zn(NO_3_)_2_ electrolytes (Fig. 10a, b), the deposited Zn–Ti anodes presented morphology of nanosheets with random orientations while the porous and interconnected frame structure of deposited Zn–Ti anodes are obtained in the Zn(CH_3_COO)_2_, ZnCl_2_ and Zn(CF_3_SO_3_)_2_ electrolytes (Fig. 10c–e). Notably, the random stacked Zn nanosheets can be a possible reason for the formation of poor electrical conductivity by-products. Furthermore, the electrically isolated property of Zn nanosheets also leads to a high resistance of electrode, which can be verified by the CEs performance displayed in Fig. 10g. The Zn–Ti nanosheets presented almost zero CE in Zn(NO_3_)_2_ electrolyte, indicating an irreversible Zn depositing/stripping behavior. The CE of Zn–Ti anode based on ZnSO_4_ electrolyte can maintain the same as the other three electrolytes at first 20 cycles, but then quickly declined to ~ 80%. Nevertheless, the CEs of Zn–Ti anodes based on the other three electrolytes are relatively stable and gradually exceeded 90%. Additionally, the impact of electrolyte concentration was investigated, for all these electrolytes, the CEs are promoted and stabilized with the increasing of electrolyte concentration. Among these electrolytes, the maximum CE of 98.4% is achieved by the 3.0 M Zn(CF_3_SO_3_)_2_ electrolyte, and the CE is saturated even further increase the concentration of Zn(CF_3_SO_3_)_2_ electrolyte. Moreover, as shown in Fig. 10h, CE of 3.0 M Zn(CF_3_SO_3_)_2_ electrolyte stably maintained for 1000 cycles, demonstrating the high reversibility of Zn depositing/stripping processes in Zn(CF_3_SO_3_)_2_ electrolyte. Comparably, saturated Zn(CH_3_COO)_2_ electrolyte also reached a high CE of ~ 95%, which enable it a potential competitor to Zn(CF_3_SO_3_)_2_ electrolyte.

Generally, ZnSO_4_ and Zn(CF_3_SO_3_)_2_ are the most frequently used electrolytes in the reported ZHSCs researches, here we chiefly discuss these two electrolytes. On the one hand, by-products and nanosheets Zn layer may cause electrically isolated Zn and increase the resistance, and then lead to the inferior CEs in ZnSO_4_ electrolytes [82]. The by-products will generate on both cathode and anode, which makes an adverse impact on the cycling performance of ZHSCs. However, the by-product can provide considerable capacity for the ZHSCs at first few cycles and the capacity in ZnSO_4_ is relatively superior to the ZHSCs in Zn(CF_3_SO_3_)_2_ electrolyte [188]. On the other hand, as for the Zn(CF_3_SO_3_)_2_ electrolyte, evidences showed that the strong interaction between water molecules and CF_3_SO_3_^−^ anions can recede the interaction between Zn-ions and adjacent water molecules, thus suppressing the formation of by-products. The utilization of Zn(CF_3_SO_3_)_2_ electrolyte can improve the reversibility of the formation process and enable the ZHSCs with a high efficiency of Zn depositing/stripping processes as well as a long cycling lifetime. However, the formation of by-products cannot be entirely bypassed even in Zn(CF_3_SO_3_)_2_ electrolytes [197]. Summarily, the ZHSCs in ZnSO_4_ electrolyte can provide higher capacity but low efficiency as well as relatively inferior longevity. The Zn(CF_3_SO_3_)_2_ electrolyte shows faster kinetics and high-efficient Zn depositing/stripping processes while it can only provide a limited capacity and its price is too expensive (> 18 times than ZnSO_4_ electrolyte) [67].

Except for the effects on the Zn anode, the anion carriers also result in a significant difference in the Cap electrodes. To investigate the effects of anions carriers on the electrochemical energy storage behaviors of Cap electrodes, Huang et al*.* applied the Zn//TiN ZHSCs as a model in three different Zn-ion salt electrolytes including ZnSO_4_, ZnAc_2_ and ZnCl_2_ [169]. After 900 cycles tests, the Zn//TiN ZHSCs in ZnSO_4_ presented the highest capacity of 405.1 F g^−1^ at a current density of 0.5 A g^−1^ among three Zn-ion salt electrolytes while the other two with ZnAc_2_ and ZnCl_2_ only show 162.3 and 245.9 F g^−1^, respectively. The capacity difference is ascribed to the adsorption energy between anions and TiN cathodes, where the SO_4_^2−^ showed a more negative adsorption energy of -6.3942 V, which is almost two times larger than Cl^−^ and Ac^−^. Moreover, the negative adsorption energy of SO_4_^2−^ can lead to a more stable structure of TiN-SO_4_ after adsorption process. As illustrated in Fig. 10i, the stable structure of TiN-SO_4_ also make a significant contribution to the anti-self-discharge ability of Zn//TiN ZHSCs. Since the Zn anode would not spontaneously convert to Zn-ions, the adsorption interaction between the anions and TiN cathode is the hinge to inhibit the self-discharge behaviors. With the stable lock of TiN-SO_4_ structure, the Zn//TiN ZHSCs offered an excellent self-discharge performance of 83.92% capacity retention after 500 h resting time.

In another work, Wang et al*.* demonstrated the great influence of water molecules on the Zn-ion electrolytes, where the diameter of Zn-ion can vastly increase from 1.48 Å (bare Zn^2+^) to 8.60 Å ([Zn(H_2_O)_6_]^2+^) [170]. The large diameter of hydrated Zn-ions may lead to a significant loss of capacity due to ion-sieving effects of porous carbon electrodes. Since the cation is Zn-ion, the anions in different Zn-ion electrolytes are crucial parts to promote the desolvation of Zn-ions and enhance the capacity of ZHSCs. Hence, Wang et al*.* investigated various Zn salt electrolytes and demonstrated that the ZnCl_2_ salt electrolyte is an optimal choice for desolvation of Zn-ions. As shown in Fig. 10j, the density functional theory (DFT) calculation results presented that the desolvation energy required by [ZnCl(H_2_O)_5_]^+^ is only half of that required by [Zn(H_2_O)_6_]^2+^, thus, the [ZnCl(H_2_O)_5_]^+^ can easily lose water molecule and get access to the micropores of carbon electrodes. Among the applied Zn salt electrolytes, the ZnCl_2_-based ZHSCs reached the highest capacity of 229.4 F g^−1^ at 1 A g^−1^. Furthermore, ZnCl_2_ electrolytes enable the smallest *R*_ct_ and the fastest ion exchange rates among various electrolytes. Notably, a remarkable cycling performance of 95.1% capacity retention was realized based on the ZnCl_2_/PAM hydrogel electrolyte after incredible 100,000 cycles, together with good low-temperature adaptability (only 7.1% capacity decay after 40,000 cycles at − 20 °C) and excellent mechanical flexibility.

By far, the most frequently used electrolytes in reported ZHSCs are ZnSO_4_ and Zn(CF_3_SO_3_)_2_, however, the dilemma of ZnSO_4_ and Zn(CF_3_SO_3_)_2_ may limit the development of high-performance ZHSCs. Here, we strongly believe that some novel electrolytes like Zn(CH_3_COO)_2_, Zn(ClO_4_)_2_ and ZnCl_2_ can be the choices to promote the combination of high-performance, long cycling lifetime and by-product-free ZHSCs. For example, Xu et al*.* reported Zn//1.0 M Zn(CH_3_COO)_2_ //rGO/PPD ZHSCs with an ultra-high areal capacity of ~ 3 F cm^−2^ at a current density of 1 mA cm^−2^, and outstanding cycling stability which maintains 100% of initial capacity after 4000 cycles [140]. Additionally, Yin et al*.* assembled the Zn//PC800 ZHSCs in the 3.0 M Zn(ClO_4_)_2_ electrolyte, presenting a wide operating voltage window (0–1.9 V) and prominent capacity of 179.8 mAh g^−1^ as well as superb capacity retention of 99.2% after 30,000 cycles [108]. Moreover, Li et al. prepared the Zn-Ti_3_C_2_T_*x*_//Ti_3_C_2_T_*x*_ ZmSCs in 6.0 M ZnCl_2_ gel electrolyte, and delivered a remarkable cycling performance of 79.6% capacity retention after 50,000 cycles [153]. Therefore, more attentions should be focused on these novel electrolytes and the interactions between the electrolyte anions and both electrodes, which may promote the realization of the balanced, high-performance and high-efficient ZHSCs.

### Quasi-Solid-state Electrolyte

With the rapid development of wearable electronic devices, flexible ESSs are in great demands. As the most important part of flexible ESSs, the electrolyte featured with flexibility, high mechanical strength, excellent wettability and high safety can enable flexible ESSs with the same properties as the aqueous systems. However, the relatively low electrical conductivity is still an obstacle for high-performance flexible devices. Due to the fast kinetic of ZHSCs, the solid-state electrolyte with insufficient conductivity is inappropriate for flexible ZHSCs devices. Instead, the quasi-solid-state or gel electrolytes are suitable candidates for flexible ZHSCs devices. Typically, the gel electrolytes of reported flexible ZHSCs include the zinc salts (ZnCl_2_ [34, 153], ZnSO_4_ [77], etc.), additive (MnSO_4_ [34], ZnBr_2_ [198], etc.), polymeric frameworks (PVA [85], PAA [120], PAM [99], gelatin [83, 96], etc.) and a small amount of water. To some extent, the gel electrolytes can suppress some side effects, such as the dissolution of electrodes, Zn dendrite and water decomposition [67]. Notably, ethylene glycol (EG), as a common used antifreeze, was applied as an additive in the quasi-solid-state electrolyte for anti-freezing and low-temperature-tolerant flexible ZHSCs, owing to the high boiling point, high dielectric constant and splendid solubility in water [197]. The reported durable, flexible ZHSCs devices can incredibly work at ultra-low temperatures from − 15 to − 40 °C [199, 200].

### Additive Electrolyte

**Fig. 11:**
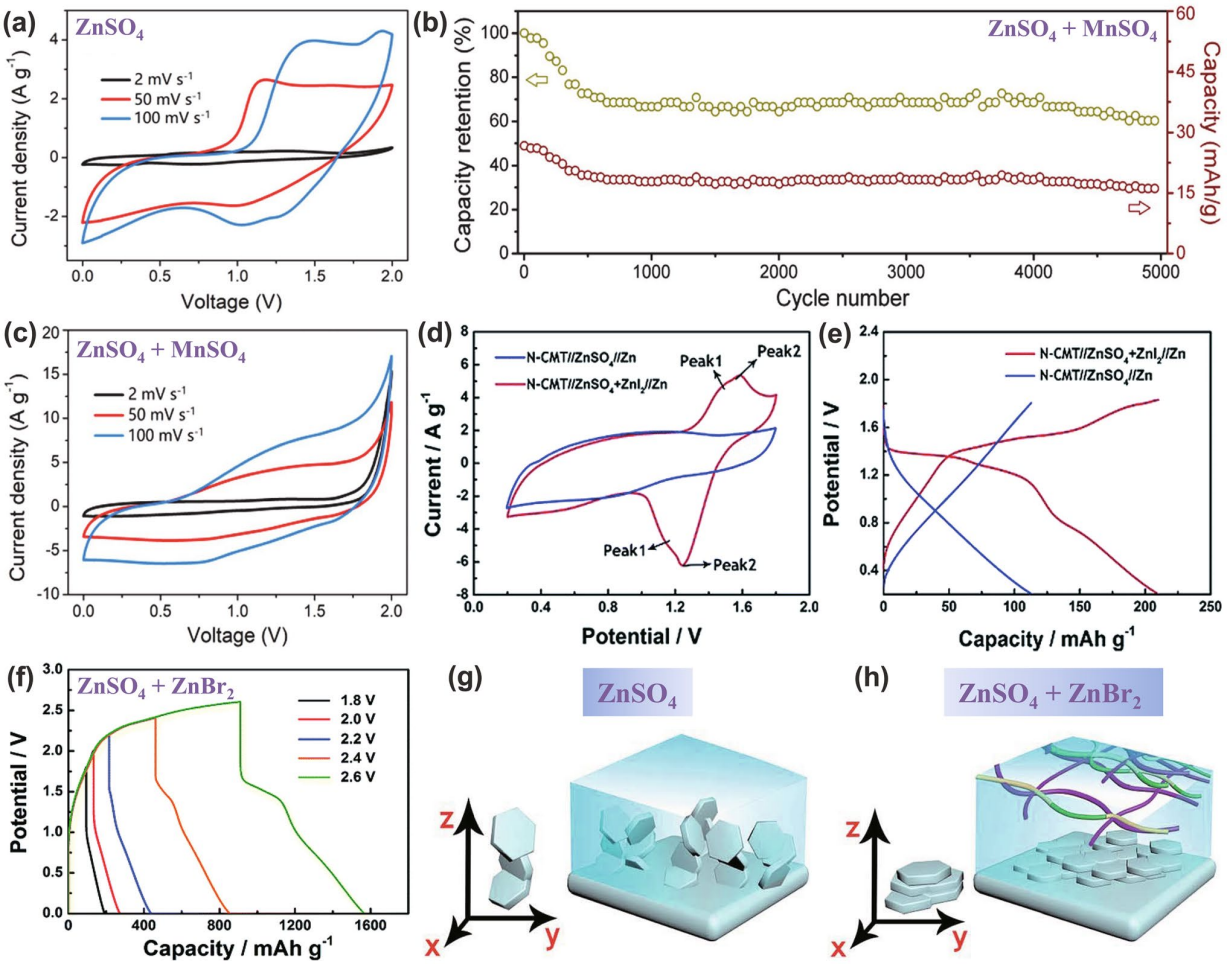
**a** CV curves of AC//2.0 M ZnSO_4_//*γ*-MnO_2_ ZHSCs. **b** Cycling performance and **c** CV curves of AC//2.0 M ZnSO_4_ + 0.5 M MnSO_4_//*γ*-MnO_2_ ZHSCs. Reproduced with permission from Ref. [188]. Copyright 2019, Elsevier. **d** CV curves and **e** GCD profiles of Zn//N-CMT ZHSCs in different electrolytes. Reproduced with permission from Ref. [110]. Copyright 2019, Royal Society of Chemistry. **f** GCD curves with diverse voltage windows. **g**, **h** Schematic illustration of Zn deposition in **g** ZnSO_4_ and **h** SA-Zn-Br hydrogel electrolytes. Reproduced with permission from Ref. [198]. Copyright 2020, Royal Society of Chemistry

Additive electrolytes are the common strategies to further stabilize or promote the electrochemical performance of many ESSs [201, 202]. With the synergy effects of various cations or anions, the energy storage behaviors are modified, and then excellent electrochemical performances can be obtained. Among the numerous additive electrolytes, MnSO_4_ is a superb choice for the stabilization of MnO_2_ in ZIBs [67]. For instance, Kang group investigated the effect of MnSO_4_ additive electrolyte in the AC//*γ*-MnO_2_ ZHSCs system [188]. Figure 11a exhibits the asymmetric CV curves of AC//*γ*-MnO_2_ ZHSCs in 2.0 M ZnSO_4_ electrolyte, indicating the typical Zn-ion insertion/desertion redox reactions of *γ*-MnO_2_ cathode. Due to the high dissolvability and poor electrical conductivity of manganese-based materials, the AC//*γ*-MnO_2_ ZHSCs can merely keep 65.3% of initial capacity after short 3000 cycles without the MnSO_4_ additive electrolyte. However, when applied the 0.5 M MnSO_4_ as the additive electrolyte, the addition of Mn^2+^ provided a positive effect on the cycling performance, the ZHSCs presented a retention of 61.8% after prolonged 5000 cycles where the capacity decay quite slow after the first 500 cycles (Fig. 11b). After first 500 cycles, the capacity retention was measured to be ~ 88% for 4500 cycles. In addition, the ACC//Zn_*x*_MnO_2_ ZHSCs assembled by Mai group can even achieve 83.1% capacity retention after 5000 cycles based on 0.4 M MnSO_4_ additive electrolyte [34].

Hence, it is believed that the utilization of MnSO_4_ additive electrolyte can effectively restrain the dissolution process of MnO_2_ by equilibrating the Mn element between MnO_2_ electrodes and electrolytes [203]. Additionally, as exhibited in Fig. 11c, the Zn-ion energy storage behavior of *γ*-MnO_2_ electrodes is changed due to the addition of Mn^2+^, the CV curves become more symmetrical and distinct polarization peaks are observed near to 2.0 V. Compared with the non-Mn^2+^ ZHSC system, the capacity of AC//*γ*-MnO_2_ ZHSCs in the mixed electrolyte of 2.0 M ZnSO_4_ and 0.5 M MnSO_4_ was improved from 54.1 to 83.8 mAh g^−1^. Many reported studies demonstrated that the Mn^2+^ of the MnSO_4_ additive electrolyte also take part in the energy storage reactions of MnO_2_ cathode. By oxidizing to MnO_2_ and further depositing on the surface of MnO_2_ cathode, the Mn^2+^ gradually become the additional host electrode for Zn-ion storage [203, 204].

As we reviewed in Sect. 4.1, the significant difference of electrolyte anions may cause distinct energy storage behaviors of ZHSCs. Therefore, the anion additive electrolyte can be a big variate for enhancing the electrochemical performance of ZHSCs. Han et al*.* applied 0.01 M zinc iodide (ZnI_2_) as the additive electrolyte, then the as-prepared Zn//N-CMT ZHSCs delivered a remarkable capacity of 416.6 mAh g^−1^ at 1 A g^−1^, which is almost two times higher than the capacity of I^−^-free ZHSCs [110]. As shown in Fig. 11d, the CV curves present diverse shapes of Zn//N-CMT ZHSCs in the ZnSO_4_ and ZnSO_4_ + ZnI_2_ electrolytes. Without the ZnI_2_ additive electrolyte, the Zn//N-CMT ZHSCs showed distorted rectangular CV curve and no redox peak, indicating the leading role of EDLC. When employed the ZnI_2_ additive electrolyte, the evident redox peaks can be detected at approximately 1.2 and 1.5 V. Typically, each peak contains two tiny peaks where the peak 1 refers to the reaction of 2I^−^/I_2_ and the peak 2 correspond to the reaction of 3I^−^/I_3_^−^ [205, 206]. The energy storage reactions are enriched due to the addition of I^−^ and the redox reactions of I^−^ generally occur on the surface of N-CMT. In addition, I^−^ added ZHSCs exhibited obvious charging/discharging platforms at ~ 1.4 V (Fig. 11e). A small amount of I^−^ additive can tremendously enhance the capacity of ZHSCs but not sacrifice the power performance, the Zn//BN-CMTs ZHSCs with I^−^ additive can offer the second-highest energy density of 472.6 Wh kg^−1^ at an incredible power density of 1600 W kg^−1^. Also, providing a peak power density of 16,000 W kg^−1^ at high current density. The redox reaction equations of iodide are presented as following Eqs. (4-1, 4-2) [198]:

4-1 $${\text{I}}_{{2}} + {\text{ 2e}}^{ - } \leftrightarrow {\text{ 2I}}^{ - }$$

4-2 $${\text{I}}_{{3}}^{ - } + {\text{ 2e}}^{ - } \leftrightarrow {\text{ 3I}}^{ - }$$

Subsequently, by further tuning of halogen elements, Han et al*.* replaced the ZnI_2_ additive electrolyte with zinc bromide (ZnBr_2_), then the highest energy density and ultra-high voltage window of 2.6 V as well as controllable Zn deposition of Zn//AC ZHSCs were achieved [198]. Sodium alginate (SA), ZnSO_4_ and ZnBr_2_ were employed to form the SA-Zn-Br hydrogel electrolyte. Unlike the symmetrical CV curve of I^−^ added ZHSCs, the Br^−^ added ZHSCs presented evident polarizations. The asymmetric CV indicated the irreversible redox reactions of Br^−^ added ZHSCs. Moreover, the Br^−^ added flexible ZHSCs devices kept a capacity retention of 87.7% after 5000 cycles, which is inferior to the 99.3% capacity retention of I^−^ added ZHSCs after 10,000 cycles. Even though, the Br^−^ additive electrolyte provided ZHSCs with the ultra-high voltage windows above normal 1.8 V, as depicted in the GCD plots of Fig. 11f. Although the CE of ZHSCs signally decreased due to the over-high voltage of 2.6 V, the charging/discharging behavior can be balanced at 2.4 V, which is still much higher than reported ZHSC systems. On account of the ultra-high voltage window, the assembled Br-added ZHSCs offered a maximum energy density of 605 Wh kg^−1^ at the power density of 1848 W kg^−1^. The energy density will decrease to 390.9 Wh kg^−1^ when lower the voltage window to 2.4 V. More importantly, the application of SA-Zn-Br electrolyte enabled the Zn deposition more controllable. As reviewed in Sect. 4.1, Zn is deposited as the nanosheets with random orientations in ZnSO_4_ electrolytes, where the schematic is illustrated in Fig. 11g. However, due to the interactions between charged groups and Zn-ions, Br^−^ can efficiently harmonize the migration of Zn-ion with uniform nucleation on Zn foil anodes [207, 208], and serve as an inhibitor of water/oxygen to avoid the corrosion of Zn foils. Therefore, as depicted in Fig. 11h, layered Zn depositions are controlled by the Br^−^ additive electrolyte. Similarly, the Br^−^ redox reaction equations are presented as following Eqs. (4-3, 4-4) [198]:

4-3 $${\text{Br}}_{{2}} + {\text{ 2e}}^{ - } \leftrightarrow {\text{ 2Br}}^{ - }$$

4-4 $${\text{2Br}}_{{2}} + {\text{ 2e}}^{ - } \leftrightarrow {\text{ Br}}_{{3}}^ {- } + {\text{ Br}}^{ - }$$

## Zn-Ion Hybrid Micro-Supercapacitors

Apart from aqueous ZHSCs system, many high-performance ZmSCs have been realized based on the in-plane electrochemical energy storage [32, 97, 153, 209, 210]. By far, only the electrode configuration of Zn//Cap ZmSCs are reported. Notably, the reported ZmSCs can achieve higher capacities than most traditional micro-supercapacitors (mSCs), including symmetric/asymmetric micro-supercapacitors and novel Li-ion micro-supercapacitors (SmSCs, AmSCs and LmSCs) [211–219]. Table 3 summarized the electrochemical performance of various reported micro-supercapacitors. Besides the capacity, the overall performances of ZmSCs is superior to other mSCs devices.

**Table 3:** Summary of electrochemical performance for the reported ZmSCs, SmSCs, AmSCs and LmSCs

Cathode	Anode	Capacity (mF cm^−2^)	Current density	Energy density (μWh cm^−2^)	Peak power density (mW cm^−2^)	Cycle	Cycle current density	Retention (%)	Voltage (V)	Electrolyte	Type	References
CNT	Recoverable Zn	83.2	1 mA cm^−2^	29.6	0.8	6000	5 mA cm^−2^	87.4	0.2–1.8	1.0 M ZnSO_4_/gelatin gel	ZmSCs	[97]
AC	Zn nanosheets	1297	0.16 mA cm^−2^	115.4	32	10,000	1.56 mA cm^−2^	100	0.5–1.5	2.0 M ZnSO_4_	ZmSCs	[32]
Ti_3_C_2_T_*x*_	Zn/Ti_3_C_2_T_*x*_	72	10 mV s^−1^	20	5.0	50,000	–	79.6	0–1.4	6.0 M ZnCl_2_/PVA gel	ZmSCs	[153]
MXene	MXene	27.3	0.02 V s^−1^	2.34	0.09	10,000	0.05 V s^−1^	100	0–0.6	1.0 M H_2_SO_4_/PVA gel	SmSCs	[217]
rGO/V_2_O_5_	rGO/V_2_O_5_	24	0.005 V s^−1^	3.3	0.11	10,000	–	94	0–1.0	3.0 M H_3_PO_4_/PVA gel	SmSCs	[211]
VN^a^	VN	40	9.5 mA cm^−2^	2	10	3000	20 mA cm^−2^	80	0–0.6	1.0 M KOH	SmSCs	[214]
MnO_2_	MnO_2_	4.31	0.05 mA cm^−2^	0.38	–	1000	0.3 mA cm^−2^	73	0–0.8	1.0 M Na_2_SO_4_	SmSCs	[219]
MnO_2_	GQDs	1.11	0.015 mA cm^−2^	0.154	0.00751	5000	1 V s^−1^	93	0–1.0	0.5 M Na_2_SO_4_	AmSCs	[212]
GQDs^b^	PANI	0.21	0.015 mA cm^−2^	0.029	0.000746	1500	1 V s^−1^	86	0–1.0	0.8 M H_3_PO_4_/PVA gel	AmSCs	[216]
Co(OH)_2_	rGO	2.28	0.05 mA cm^−2^	0.35	0.1	10,000	0.49 mA cm^−2^	89	0–1.4	1.3 M KOH/PVA gel	AmSCs	[218]
VO_*x*_/rGO	VN/rGO	207.9	0.63 mA cm^−2^	73.9	3.77	8000	6.0 mA cm^−2^	65	0–1.6	5.0 M LiCl/PVA gel	LmSCs	[215]
Graphene-MnO_2_	Graphene-FeOOH	21.6	0.25 mA cm^−2^	9.6	11.85	2000	1.0 mA cm^−2^	84	0–1.8	4.7 M LiCl/PVA gel	LmSCs	[213]

^a^*VN* vanadium nitride

^b^*GQDs* graphene quantum dots

**Fig. 12:**
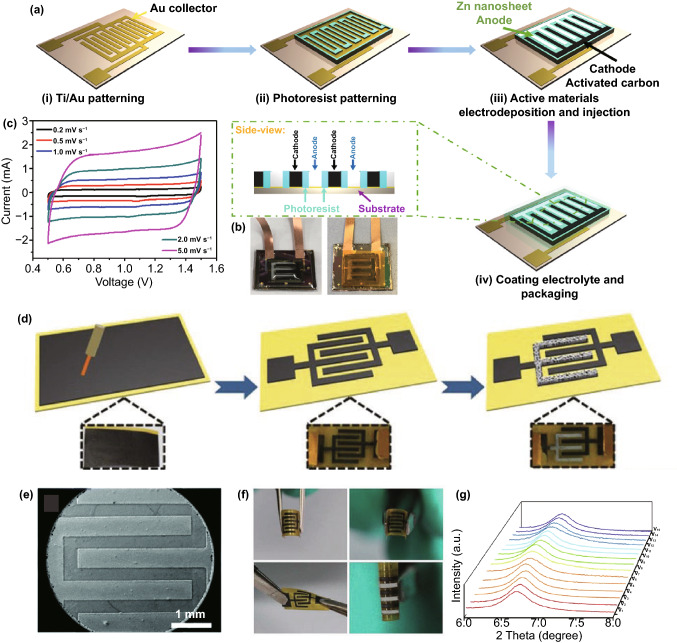
**a** Fabrication process, **b** optical images before and after packaging and **c** CV curves of the Zn//AC ZmSCs. Reproduced with permission from Ref. [32]. Copyright 2019, Wiley–VCH. **d** Preparation processes of the Zn//CNT ZmSCs. **e** SEM image of microelectrode. **f** Photographs of the Zn//CNT ZmSCs at the bent and twisted states. Reproduced with permission from Ref. [97]. Copyright 2018, Royal Society of Chemistry. **g** Ex-situ XRD spectra of Ti_3_C_2_T_*x*_ cathode in ZmSCs. Reproduced with permission from Ref. [153]. Copyright 2021, Springer Nature

For one, Feng group fabricated ZmSCs where the electrodeposited Zn nanosheets act as the anodes and the AC on the opposite electrode act as cathodes in the ZnSO_4_ aqueous electrolyte [32]. Figure 12a exhibits the standard lithography processes involved in the Zn//AC ZmSCs, and the optical images of as-assembled ZmSCs before and after packaging are shown in Fig. 12b. Remarkably, the Zn//AC ZmSCs provided an ultra-high areal capacity of 1297 mF cm^−2^ at 0.16 mA cm^−2^, which is almost 5–500 times higher than SmSCs and AmSC. Meanwhile, the energy density of the ZmSCs reached 115.4 μWh cm^−2^ at a power density of 0.16 mW cm^−2^. In addition, it presented an ultra-stable cycling performance with 100% capacity retention after 10,000 cycles at 1.56 mA cm^−2^, which can be further confirmed by the symmetric rectangular shape of CV curves (Fig. 12c) at various scan rates of 0.2–5 mV s^−1^. For another, Sun et al*.* assembled Zn//CNT ZmSCs with recoverable Zn anodes and the fabrication processes are shown in Fig. 12d [97]. The CNT paper was firstly pasted onto the polyimide tape and then patterned interdigital microelectrodes employing a computer-controlled laser cutting system. The SEM image of microelectrode is displayed in Fig. 12e. Typically, the measured areal energy density of the Zn//CNT ZmSCs is 29.6 μWh cm^−2^ and the peak power density is 8 mW cm^−2^. Furthermore, the flexible ZmSCs devices are also investigated by introducing the ZnSO_4_ gel electrolyte, the digital photos of Zn//CNT ZmSCs devices at the bent and twisted states are depicted in Fig. 12f, indicating the splendid mechanical flexibility of flexible ZmSCs devices. Remarkably, the ZmSCs can offer stable GCD curves at different degrees of inflection and maintained excellent capacity retention of 89.5% after 1000 mild bending cycles. Lately, Shen group applied 2D layered Ti_3_C_2_T_*x*_ as the cathode of ZmSCs and realized great longevity of 50,000 cycles, keeping a capacity retention of 79.6% [153]. Significantly, the energy storage mechanisms of as-prepared ZmSCs were investigated by ex-situ XRD spectra shown in Fig. 12g, the characteristic peak (002) of Ti_3_C_2_T_*x*_ shifted to the left by degree during discharging processes. According to Bragg’s law (2*d*sin*θ* = *nλ*), the decrease of the peak (002) degree refers to the increase of interlayer spacing due to Zn-ion insertion. On the contrary, the peak gradually moved to the initial degree during the charging process. Therefore, the Zn-ions insertion/desertion processes display similar energy storage behaviors as aqueous ZHSCs.

With the miniaturization of electronic devices, the demands for micro electronic devices are growing, like wireless headsets and micro-ESSs. Therefore, it is believed that the study of ZmSCs will become a hot spot and bring a thorough revolution to mSCs research area. However, up to now, the studies of ZmSCs are lacking and short of an in-depth understanding of the unique in-plane energy storage behaviors. From our point of view, we deem that more suitable electrode materials should be attempted for ZmSCs to form an optimal configuration and make full use of the limited space of ZmSCs.

## Flexible Zn-Ion Hybrid Supercapacitors

Emerging flexible electronic devices are well developed in recent years, such as smart textiles, soft robotics and bio-sensors [220–223]. Further paradigm shift should be the seamless integration of flexible devices with flexible ESSs. Among the Zn-ion energy storage systems, flexible ZIBs have been widely investigated owing to various features of high flexibility, superb mechanical strength, excellent stability and safety [86, 224]. The application of gel and even solid-state electrolyte make the flexible ZIBs as a promising candidate for the flexible energy storage systems. However, most reported flexible ZIBs suffer from the short longevity (about 100 to 1000 cycles) and low power density [225–227]. Under this circumstance, the discovery of ZHSCs aroused the interests of flexible ZHSCs principally due to a range of attractive features, such as ultra-long cycling lifetime, cost-effectiveness, good safety and eco-friendliness. By far, two typical types (sheet-type and fiber-type) of flexible ZHSCs have been reported, to some extent, the shapes of the flexible ZHSCs can determine the corresponding conditions of practical application, for instance, wearable electronic devices usually demand high areal capacity flexible ZHSCs, which can minimize the thickness of the devices and create a better wearing feeling. When applied to the wireless headset, the 1D fiber-shape ZHSCs are preferred due to the 3D flexibility of the linear structure. In the following section, we amply summarized the components and structure of the two typical types of flexible ZHSCs.

### Sheet-Type ZHSCs

**Fig. 13:**
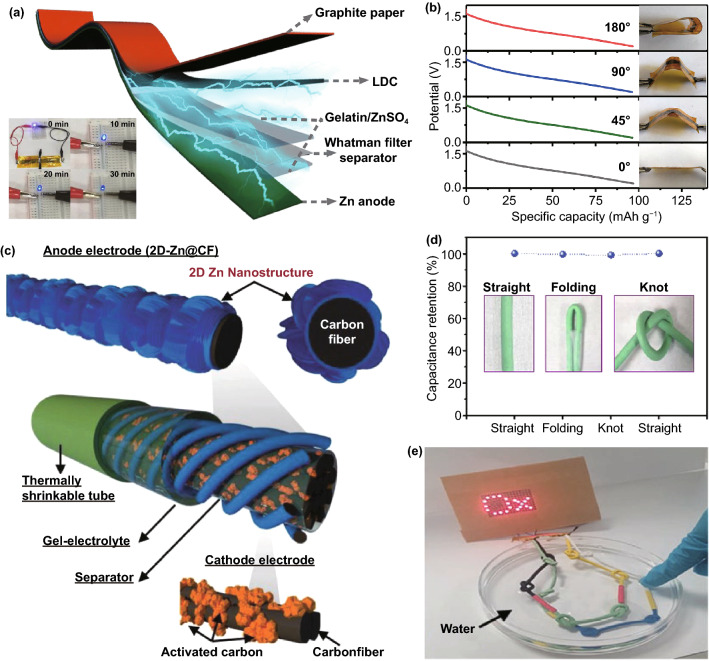
**a** Schematics of sheet-type flexible ZHSCs. **b** Discharge curves under different deformation conditions. Reproduced with permission from Ref. [83]. Copyright 2019, Elsevier. **c** Schematic of fiber-type flexible ZHSCs. **d** Capacity retention under the condition of straight, folded and knotted. **e** LED powered by connected fiber-type ZHSCs in water. Reproduced with permission from Ref. [53]. Copyright 2020, Wiley–VCH

Sheet-type flexible ZHSCs focus on promoting the areal capacity depends on sheet-type flexible electrodes. Up to now, many outstanding investigations have been reported [99, 132, 154, 198]. For one, Lu et al*.* built a quasi-solid-state Zn//ZnSO_4_ (gel)//BN-LDC sheet-type flexible Zn//Cap ZHSCs, the schematic illustration is depicted in Fig. 13a where the graphite paper acted as the current collection of LDC cathode, the Zn foil served as anode and current collector [83]. Moreover, the Whatman filter and 1.0 M ZnSO_4_ gel are employed as separator and electrolyte, respectively. Correspondingly, the sheet-type ZHSCs provided a specific capacity of 116.8 mAh g^−1^ at 0.5 A g^−1^ and further retain 55.4 mAh g^−1^ at 20 A g^−1^. Additionally, the Zn//BN-LDC flexible ZHSCs device can be arbitrarily bent or twisted without deteriorating the discharge curve and capacity (97 mAh g^−1^ at 1 A g^−1^, Fig. 13b). Typically, the series-wound ZHSCs are employed in a practical application and lightened a blue light-emitting diode (LED) for ~ 30 min (inset of Fig. 13a). Notably, the flexible ZHSCs exhibited a good cycling performance of sustaining 81.3% of the initial capacitance and nearly 100% CE after 6500 cycles. The Zn foil presented no evident dendrites and the BN-LDC retains 2D layered structure after a long-term cycling test. For another, the sheet-type flexible ZHSC of Cap//ZBC ZHSCs was reported by Mai group [34]. The flexible ACC//Zn_*x*_MnO_2_ ZHSCs can afford a prominent capacity of 1446.6 mF cm^−2^ and ultra-high areal energy density of 803.6 μWh cm^−2^. Considering the above results, the 2D sheet-type flexible ZHSCs show wild application prospects for plane electronic devices due to the splendid areal electrochemical performance, good mechanical flexibility and high safety, which can be a promising candidate for future flexible ESSs.

### Fiber-Type ZHSCs

Compared with usual sheet-type flexible ZHSCs, fiber-type flexible ZHSCs are less discussed, however, the fiber-type ZHSCs based on one-dimensional (1D) fiber-shape electrodes can perform better mechanical flexibility than 2D sheet-type ZHSCs. Typically, the flexibility of fiber-type ZHSCs is based on the flexible fiber substrate such as carbon fiber (CF), graphite fiber and CNT [120], while the sheet-type ZHSCs achieve flexibility by the intrinsic flexible electrodes like Zn foil and CC [34, 83]. An et al*.* prepared an outstanding 1D fiber-type ZHSCs and the explicit schematic illustration is depicted in Fig. 13c [53]. Structurally, the fiber-type ZHSCs are composed of 2D-Zn@CF anode, AC@CF cathode, separator, gel electrolyte and thermally shrinkable tube. The AC@CF cathode is enclosed by cellulose papers which act as the separator. Subsequently, the AC@CF cathode is weaved by the 2D-Zn@CF anode and the 1.0 M ZnSO_4_/PVA gel electrolyte is coated on the fiber-type ZHSCs, then covered with thermally shrinkable tubes to protect fiber-type ZHSCs from external influence. The length and diameter of the fiber-type ZHSCs are 15 cm and 8–9 μm, respectively. Based on the well-designed structure, the fiber-type ZHSCs provided a high capacity of 56–24 mF cm^−2^ at a current density of 0.05–3.0 mA cm^−2^. Moreover, the energy density of 25 μWh cm^−2^ is realized by fiber-type ZHSCs at a high power density of 50 μW cm^−2^. As shown in Fig. 13d, the fiber-type ZHSCs can keep the initial energy storage performance under the condition of folding and knotting, demonstrating superb mechanical flexibility of fiber-type ZHSCs. To evaluate the waterproof capability of the fiber-type ZHSCs, the LEDs were powered by multiple fiber-type ZHSCs which are strongly knotted and under water immersion (Fig. 13e). Furthermore, the cycling lifetime of fiber-type ZHSCs reached 10,000 cycles, and the capacity presented no evident decay, which is superior to many reported flexible supercapacitors [228–233]. Here, we summarized the electrochemical performance of two types of flexible ZHSCs in Table 4.

**Table 4:** Electrochemical performance summary of sheet-type and fiber-type ZHSCs

Cathode	Anode	Capacity	Current density	Energy density	Peak power density	Cycle	Cycle current density	Retention (%)	Voltage (V)	Electrolyte	Device type	References
BN-LDC	Zn foil	127.7 mAh g^−1^	0.5 A g^−1^	97.6 Wh kg^−1^	12,100 W kg^−1^	6500	5 A g^−1^	81.3	0.2–1.8	1.0 M ZnSO_4_/gelatin gel	Sheet-type	[83]
HNPC	Zn foil	148.2 mAh g^−1^	4.2 A g^−1^	91.8 Wh kg^−1^	27,600 W kg^−1^	–	–	–	0–1.8	1.0 M ZnSO_4_/PVA gel	Sheet-type	[85]
HCSs	Zn/CC	86.8 mAh g^−1^	0.5 A g^−1^	59 Wh kg^−1^	447.8 W kg^−1^	15,000	1 A g^−1^	98.0	0.15–1.95	0.08 M ZnSO_4_/PAM gel	Sheet-type	[99]
Zn_*x*_MnO_2_	ACC	1446.6 mF cm^−2^	1 mA cm^−2^	803.6 μWh cm^−2^	10.0 mW cm^−2^	–	–	–	0–2.0	2.0 M ZnCl_2_ + 0.4 M MnSO_4_/PVA gel	Sheet-type	[34]
NO-BPC^a^	Zn/CC	48 mAh g^−1^	0.2 A g^−1^	35.9 Wh kg^−1^	993.4 W kg^−1^	2500	2 A g^−1^	100.0	0.2–1.8	2.0 M ZnSO_4_/PVA gel	Sheet-type	[115]
Ti_3_C_2_	Zn/Ti_3_C_2_	132 F g^−1^	0.5 A g^−1^	–	–	1000	3 A g^−1^	82.5	0.1–1.35	1.0 M ZnSO_4_/gelatin gel	Sheet-type	[96]
PDA/PCC	Zn/CC	0.92 mAh cm^−2^	2 mA cm^−2^	9.7 mWh cm^−3^	52 mW cm^−3^	500	–	92.0	0.1–1.8	2.0 M ZnSO_4_/PVA gel	Sheet-type	[154]
TDP/AC^b^	Zn foil	1.16 mAh cm^−2^	1 mA cm^−2^	1.03 mWh cm^−2^	9.0 mW cm^−2^	2000	8 mA cm^−2^	71.0	0.1–1.9	2.0 M ZnSO_4_/gelatin gel	Sheet-type	[172]
AC	Zn foil	654.8 mAh g^−1^	2 A g^−1^	605 Wh kg^−1^	–	5000	6 A g^−1^	87.7	0–2.6	~ 1.0 M Zn^2+^ (ZnSO_4_ + ZnBr_2_) /PAA-AM gel	Sheet-type	[198]
rGO/CNT	Zn/G	104.5 F cm^−3^	400 mA cm^−3^	48.5 mWh cm^−3^	3.6 W cm^−3^	10,000	3.2 mA cm^−3^	98.5	0–1.8	2.0 M ZnSO_4_/PAA gel	Fiber-type	[120]
rGO/CNT	Zn/G	84.7 F cm^−3^	400 mA cm^−3^	–	–	10,000	3.2 mA cm^−3^	86.2	0–1.8	2.0 M ZnSO_4_/PVA gel	Fiber-type	[120]
AC	2D-Zn/Ni	56 mF cm^−2^	0.05 mA cm^−2^	25 μWh cm^−2^	3.0 mW cm^−2^	10,000	1 mA cm^−2^	100.0	0.2–1.8	1.0 M ZnSO_4_/PVA gel	Fiber-type	[53]

^a^*NO-BPC* N, O co-doped bamboo porous carbon

^b^*TDP/AC* poly(4,4’-thiodiphenol, TDP)/AC

According to the above reviews and our own experience, the keys to fabricating a flexible ZHSC are gel electrolyte and packaging technology. During the fabrication processes, the flexible devices tend to short-circuit due to the fast diffusion rate and electron transport, which can lead to a high defect rate. A great number of electrolytes are added to achieve excellent contact between electrodes and electrolytes, but the flow of the quasi-solid-state is uncontrollable. In this regard, short-circuit can quickly occur when the separator fails due to the leakage of electrolyte or some manufactural defects of the separator. In addition, the flexible ZHSCs can maintain stable charging/discharging processes at low current density, however, turn to short-circuits at high current density. Once the short circuits occur, the flexible ZHSCs devices cannot return to normal even at low current density. Hence, a relatively low concentration electrolyte and suited polymer gel may be optimal for flexible ZHSCs, Chen et al*.* employed a low concentration of 0.08 M ZnSO_4_/PAM gel electrolyte for ZHSCs, the ZHSCs presented capacity of 86.8 mAh g^−1^ and 15,000 cycles with 98% capacity retention [99]. Additionally, outstanding packaging technology can vastly decrease the probability of malfunction, for instance, the fiber-type flexible ZHSCs are preferable to sheet-type flexible ZHSCs because the electrodes are roundly covered by a separator to avoid the leakage of electrolytes, which suppresses unexpected short circuits [53]. Based on the splendid properties of ZHSCs, the flexible ZHSCs also showed unprecedented electrochemical performance among various flexible ESSs. Even though flexible ZHSC is still in its infancy and more efforts should be devoted to improving the reliability of flexible ZHSC devices, it is believed that flexible ZHSCs can be qualified for high-performance flexible electronic devices.

## Summary and Outlook

In this review, we have expounded the design theory by summarizing recent research progresses of ZHSCs and classifying them into two electrode configurations of Zn//Cap ZHSCs and Cap//ZBC ZHSCs. All in all, these ZHSCs integrate various advantages of conventional supercapacitors and ZIBs, presenting high energy density, high power density, splendid rate performance and excellent cycling longevity. In addition to the aqueous ZHSCs, this review summarizes practical applications of ZHSCs, such as high-performance ZmSCs and flexible sheet/fiber-type ZHSCs, which indicate the promising prospects of ZHSCs for various applications. Although many improvements have been achieved in the ZHSCs field, ZHSCs system is still in its infancy compared to some mature ESSs. Therefore, we believe that it should pay more attention to the following key points for the prospective research of ZHSCs:

1. *Better understandings of electrode materials are required*
  When simply employing commercial Zn foil and AC materials as electrodes, the electrochemical performances of Zn//Cap ZHSCs are relatively limited. In this regard, the development of specific electrodes for ZHSCs is quite needed, where the Zn anode should be hierarchically designed including high-performance background Zn and surface Zn while more strategies should be applied to promote the combination of carbon-based materials and some novel materials, which possess fast and pseudocapacitive Zn-ion storage mechanisms. In a word, preparing the battery-type electrodes in the methodology of Cap electrodes, while the Cap electrodes the methodology of battery-type, then the trade-off between energy density and cycling performance can be well-balanced.
2. *Attempt of mature ZBC for ZHSCs*
  Even though the Cap//ZBC ZHSC is still in its infancy, the great designability and unique properties like high voltage window and Zn-dendrite-free of Cap//ZBC ZHSCs make it a promising ESS in the future. To date, the ZBC are suffering from one or more of the following deficiencies: difficulty in precise control, inferior rate performance, weak cycling performance, low energy density. Designing advanced cathodes (e.g., Mn, V, PBA, Mo, Co-based cathodes) with superb structural stability, large specific capacity and suitable Zn-ion storage mechanisms can greatly promote the electrochemical performance of Cap//ZBC ZHSCs. Some splendid strategies for cathode materials in the ZIBs can also be applied in ZHSCs and further summarize the ZHSCs-proper design strategies of ZBC. We strongly believe that the Cap//ZBC ZHSCs deserve more investigations.
3. *Confirm the by-products mechanisms and find solutions*
  Although the precipitation/dissolution processes of Zn_4_SO_4_(OH)_6_·nH_2_O or ZnSO_3_·2.5(H_2_O) by-products have been analyzed at length in this review, more studies are needed to confirm the conclusions and find feasible ways out to prevent the irreversible precipitation/dissolution processes of both electrodes in ZHSCs. It is believed that the novel electrolytes like Zn(CH_3_COO)_2_, Zn(ClO_4_)_2_ and ZnCl_2_ will be the key for achieving high-performance and by-product-free ZHSCs.
4. *Appropriate additives may optimize energy storage behaviors*
  According to the reported works, the additive electrolytes can tremendously promote the electrochemical performance of ZHSCs. For example, the Mn-ion additive can restrain the dissolution of manganese-based materials, the Br-ion additive can increase the voltage window to 2.6 V and enable a controllable Zn deposition behavior. A few additives may vastly boost the electrochemical performance of ZHSCs, thus, more cation and anion additive electrolytes should be tried and introduced into the normal Zn-ion salt electrolytes to achieve high-performance ZHSCs with special properties.
5. *Attempts of 3D materials and establishment of mechanical standards in ZmSCs*
  The reported in-plane ZmSCs have gained much attention due to their efficient energy storage capacity, which is much more than the traditional mSCs. However, the electrodes of ZmSCs are based on thin-film electrodes and the materials are still conventional. The 3D porous Cap cathodes should be introduced for high-performance ZmSCs and some efforts are necessary for investigating the Cap//ZBC ZmSCs. Moreover, the “flexibility” of ZmSCs is still a vague definition, it is urgently needed to build a flexible mechanical standard for ZmSC, which can serve as the guidance for future assessment.
6. *Improve the stability of flexible ZHSCs devices*
  Same as the ZHSCs, the flexible ZHSCs are new products in flexible ESSs. With the excellent electrochemical performance and safe aqueous gel electrolytes, the flexible ZHSCs devices are a promising candidate for new generation flexible ESSs. The fiber-type ZHSCs can be knitted into fabrics, offering a viable solution toward wearable ESSs. However, flexible ZHSCs presented unstable charging/discharging behaviors at high current density, and the synthetic success rate is much lower than ZIBs devices due to the fast diffusion rate and electron transport. Thus, the explorations of flexible ZHSCs are far from satisfied, where the studies of low-concentration gel electrolyte and precise packaging technology may be the keys.

Although tremendous efforts have been made, it should be emphasized that the ZHSCs still need a long way to be applied in commercial applications. We would rather regard this review as an opening remark than a concluding remark. We believe the ZHSCs will break some current bottlenecks in the fields of energy storage and eventually, realizing better Zn-ion storage systems with balanced electrochemical performances.
